# Maternal Micronutrient Status During Pregnancy and Its Neurodevelopmental Implications for Infants in South Asia: Protocol for a Scoping Review

**DOI:** 10.2196/81592

**Published:** 2025-12-15

**Authors:** Jitender Nagpal, Swapnil Rawat, Neetu Bansal, Sarika Tyagi, Mehak Verma, Manu Mathur

**Affiliations:** 1 Sitaram Bhartia Institute of Science and Research, B-16, NRPC Colony, Block B, Qutab Institutional Area, New Delhi, Delhi, 110016, India, 91 9871799011; 2 Queen Mary University London, London, United Kingdom

**Keywords:** pregnancy, micronutrient, infants, neurodevelopment, South Asian countries, maternal nutrition, supplements in pregnancy, child development

## Abstract

**Background:**

Pregnancy is a crucial stage characterized by an increased demand for various nutrients. The role of micronutrients becomes especially important during pregnancy and infancy to support neurodevelopment. Micronutrient deficiencies are prevalent in low- and middle-income countries due to socioeconomic disparities, limited dietary diversity, and barriers to quality antenatal care. This results in women of reproductive age and developing offspring being disproportionately affected. Despite extensive research, evidence remains fragmented, leading to a lack of comprehensive synthesis.

**Objective:**

This scoping review aims to explore the existing evidence on the role of maternal micronutrient status during pregnancy influencing neurodevelopmental outcomes in infants. Additionally, it will assess the prevalence and distribution of specific micronutrient deficiencies and identify their sociodemographic determinants within South Asian countries.

**Methods:**

This scoping review uses an iterative, three-step search strategy to identify both published and gray literature. Initially, a targeted search using relevant keywords was developed for PubMed to locate studies investigating maternal micronutrient status or supplementation during pregnancy (women aged 15‐49 y) and associated neurodevelopmental outcomes in offspring up to two years of age. The search was sequentially narrowed by geographic region (South Asian countries), study design, human studies, English-language publications, and clinical trials. In the second stage, this search strategy will be adapted and implemented across additional electronic databases, including MEDLINE, Embase, Google Scholar, Cochrane Library, OpenGrey, JSTOR, and Wiley, as well as trial registries such as ClinicalTrials.gov and PROSPERO. Further, supplementary hand-searching of relevant journals will be conducted. The third step involves applying a snowballing technique to review the bibliographies of initially identified papers. Two reviewers will independently conduct study selection and data extraction using standardized forms. Quality assessment will use Joanna Briggs Institute critical appraisal checklists. Quantitative findings (study characteristics, exposure definitions, outcome measures) will be summarized with descriptive statistics and visualized in structured tables and charts, and qualitative findings will be coded inductively to develop themes pertinent to the review questions. We will integrate evidence through a convergent narrative synthesis to contextualize how maternal micronutrient deficiencies are influenced by sociodemographic factors and how these relate to infant neurodevelopment. The review will adhere to PRISMA-ScR reporting guidelines.

**Results:**

The initial database search was completed on July 3, 2025. Title and abstract screening is in progress, and final synthesis and reporting are anticipated by January 2026.

**Conclusions:**

This review aims to summarize the available evidence on maternal micronutrients and infant neurodevelopment in South Asia, identify major gaps and inconsistencies in the data, and highlight opportunities for focused research and multisectoral action. The findings will help guide the next phases of the South Asia Collaborative for Maternal Micronutrients and Infant Neurodevelopment (SACMIND) collaborative project, which will include qualitative studies, biomarker assessments, and designing interventions.

## Introduction

Pregnancy is a crucial life stage characterized by increased nutritional requirements, including both macronutrients (calories, proteins, fats) and micronutrients (including vitamins and minerals). Sufficient intake of these nutrients is essential to support the mother’s health and the growth and development of the fetus [1]. During pregnancy and early infancy, nutrients and growth factors play a central role in orchestrating development. In particular, brain development during the fetal and early postnatal period is highly sensitive to maternal nutrition, as nutrients regulate processes like cell proliferation and differentiation and neural connectivity. Although all nutrients contribute to fetal growth, some have especially critical impacts on the developing brain during these periods. For example, adequate protein and energy intake support overall fetal growth, and specific micronutrients act as cofactors in brain development pathways.

Several micronutrients are vital for fetal growth during pregnancy, each serving specific functions [2]. Folic acid (vitamin B9) helps prevent neural tube defects and supports early brain and spinal cord development. Vitamin D is important for fetal skeletal development, calcium metabolism, and immune function; a deficiency may impair bone growth and affect neurodevelopment. Iodine is essential for thyroid hormones and brain development, with deficiencies potentially leading to cognitive impairment or cretinism. Iron supports increased maternal blood volume and oxygen delivery to the fetus and is crucial for brain maturation, while deficiency is associated with low birth weight [3-6]. Other nutrients, such as zinc for cellular growth and reduced risk of preterm birth, vitamin B12 for neural development, vitamin A, and essential fatty acids, also contribute to healthy fetal growth and neurological outcomes [7].

Micronutrient deficiencies in pregnant women are a critical public health problem in low- and middle-income countries [1]. Women in low- and middle-income countries often begin pregnancy with pre-existing undernutrition or specific nutrient deficiencies, which can be exacerbated by the increased nutritional demands of gestation. It is also important to recognize that deficiencies often do not occur in isolation. In populations with limited dietary diversity, mothers may be lacking several micronutrients simultaneously. The highest prevalence of such deficiencies is reported in South Asia [8]. Population-based studies in South Asian countries, including India, Bangladesh, and Nepal, have reported deficiencies in zinc (15%‐74%), vitamin B12 (19%‐74%), vitamin E (as α-tocopherol, 50%‐70%), and folate (0%‐26%) among pregnant women [9]. Several investigators have documented the relationship between micronutrient deficiencies and subsequent neurodevelopment in infants [7,10,11], and several others have attempted to study the impact of supplementation [12-14]. The authors of this scoping review have also completed several studies in the field of micronutrient supplementation, including a recently concluded trial on B12 supplementation in pregnant women that resulted in better infant mental development [15-19]. However, a comprehensive overview is required to clarify both the existing evidence and the knowledge gaps regarding how maternal micronutrient status during pregnancy relates to offspring neurodevelopment. Toward this purpose, a preliminary database search found no current or ongoing scoping reviews specifically addressing the relationship between gestational micronutrient status and infant neurodevelopment. Two global scoping reviews were identified: one on maternal nutrition and neurodevelopment [7] and another on the role of zinc in neonatal brain growth [20]. Considering the complex interplay between maternal nutrition and infant neurodevelopment, a scoping review was considered appropriate to map the breadth, characteristics, and gaps of the evidence across diverse study designs and outcome measures. Given the heterogeneity in study designs, exposure definitions, gestational timing, biomarkers, and neurodevelopmental instruments, a meta-analytic approach was deemed premature. However, this review is intended to seed focused systematic reviews in the future—potentially micronutrient-specific and outcome-specific—where methodologically homogeneous subsections would permit quantitative synthesis and meta-analysis. This scoping review is a methodological component of the South Asia Collaborative for Maternal Micronutrients and Infant Neurodevelopment (SACMIND) multicountry collaborative project, which aims to elucidate how maternal micronutrient deficiencies influence infant neurodevelopment and identify effective strategies for tackling maternal micronutrient deficiencies across South Asian contexts.

## Methods

### Ethical Considerations

The study has been approved by the institutional ethics committee of the Sitaram Bhartia Institute of Science and Research, New Delhi, India (reference number F.1/SBISR-EC/PR 05-24).

### Overview

This scoping review will follow the methodological framework originally proposed by Arksey and O’Malley [21], with enhancements from Levac et al [22] and the Joanna Briggs Institute (JBI) methodology for scoping reviews [23]. The review will proceed through the following stages: (1) identifying the research question, (2) identifying relevant studies, (3) selecting studies, (4) charting the data, (5) collating, summarizing, and reporting the findings, and (6) consulting with relevant stakeholders. To maintain methodological rigor and promote reproducibility, the PRISMA-ScR (Preferred Reporting Items for Systematic Reviews and Meta-Analyses extension for Scoping Reviews) checklist will be used as a guiding tool [24].

### Stage 1: Identifying the Research Questions

Specifically, our scoping review aims to answer the following questions:

1. Which micronutrient deficiencies during pregnancy are associated with infant neurodevelopment?
2. What is the quality and spectrum of evidence from South Asia on the impact of maternal micronutrient deficiencies on infant neurodevelopment?
3. Which interventions have been attempted in South Asia to address maternal micronutrient deficiencies, and what is the evidence of their effectiveness on infant neurodevelopment outcomes?
4. What is the prevalence and geographic distribution of micronutrient deficiencies during pregnancy in South Asian countries, and which sociodemographic factors are associated with these deficiencies?
5. What are the existing gaps and future directions for research on maternal micronutrients during pregnancy and their impact on infant neurodevelopment in South Asia?

The research questions are described using the Population, Concept, and Context (PCC) framework [23] in Textbox 1.

Textbox 1. **Population, Concept, and Context framework.**

Population (P)

- Pregnant women (15-49 years) and their offspring (up to 2 years of age).

Concept (C)

- To explore the evidence for key maternal micronutrients during pregnancy linked to infant neurodevelopment.
- To study the prevalence of maternal micronutrient deficiencies during pregnancy and contributing factors associated with neurodevelopmental outcomes.

Context (C)

- The exploration of evidence on the association of key maternal micronutrients during pregnancy with infant neurodevelopment will be synthesized globally.
- The prevalence and contributing factors of maternal micronutrient deficiencies during pregnancy will be explored, with particular emphasis on the South Asian region.

### Stage 2: Identifying Relevant Studies

#### Search Strategy

**Figure 1.**
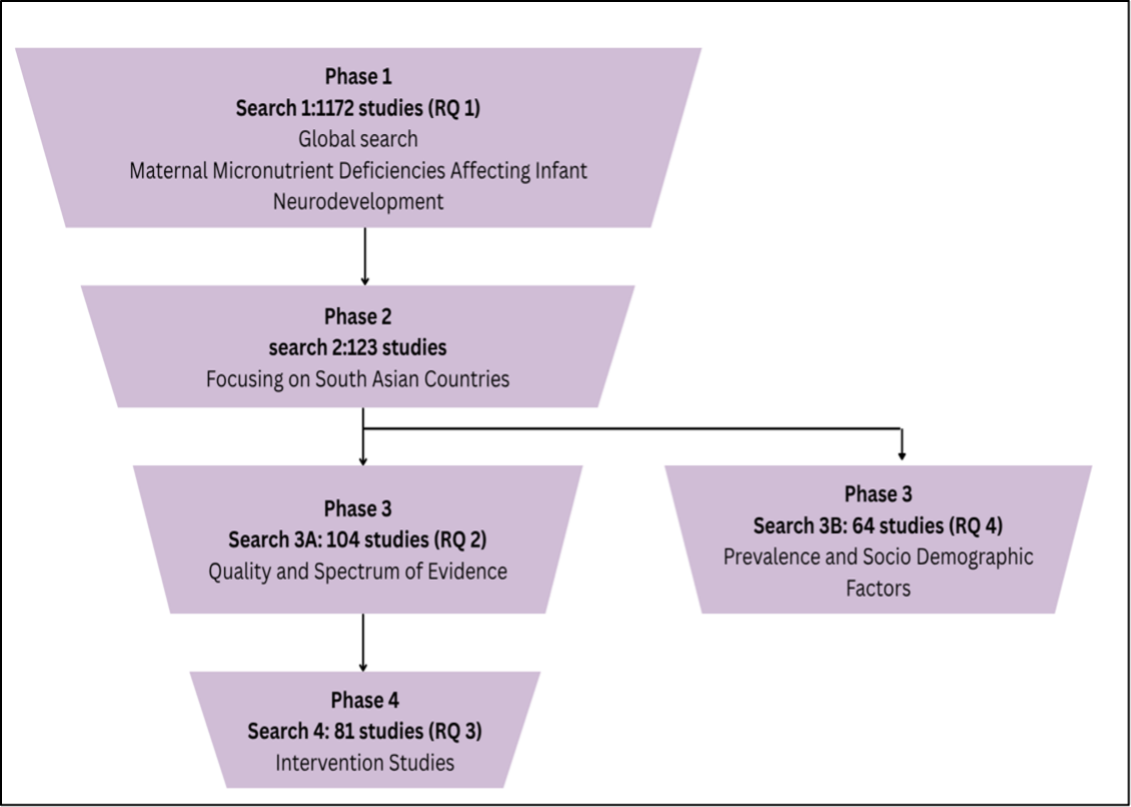
Search strategy. RQ: research question.

The three-step search strategy as prescribed by JBI will be followed to locate both published and gray literature. In Step 1, an initial search of PubMed has been undertaken. Key concepts for the search were identified; terms based on the concepts were combined using Boolean operators (OR, AND, NOT) to make queries and searched under the All Fields category. As illustrated in Figure 1, the study will be conducted in phases corresponding to the research questions (RQs). To ensure both logistical efficiency and comprehensive coverage throughout each phase, preliminary searches using the selected search terms and Boolean operators were performed on PubMed. These terms were then systematically refined for scope and practicality through an iterative process by the lead authors in consultation with all coauthors prior to finalization (Multimedia Appendix 1).

In Phase 1, a global search to identify micronutrient deficiencies during pregnancy that are associated with infant neurodevelopment was conducted. The search strategy (search 1) was developed using the following terms: ((pregnancy) OR (maternal)) AND ((infant) OR (offspring) OR (child)) AND ((micronutrient status) OR (micronutrient deficiency) OR (vitamin A) OR (vitamin B) OR (vitamin C) OR (vitamin D) OR (vitamin E) OR (vitamin K) OR (thiamin) OR (riboflavin) OR (niacin) OR (B5) OR (B6) OR (biotin) OR (folic acid) OR (cobalamin) OR (iron) OR (calcium) OR (sulfur) OR (magnesium) OR (phosphorus) OR (sodium) OR (potassium) OR (zinc) OR (copper) OR (manganese) OR (molybdenum) OR (boron) OR (chlorine) OR (chloride) OR (selenium) OR (cobalt) OR (fluorine) OR (fluoride) OR (iodine) OR (silicon)) AND ((neurodevelopment) OR (“brain development”) OR (“motor development”) OR (“psychomotor development”) OR (“language development”) OR (“cognitive development”) OR (cognition)) NOT (toxic) NOT (gene) NOT (autism) NOT (preterm). This global search yielded 1172 studies.

In Phase 2, the search strategy was narrowed down (search 2) to focus on studies conducted in South Asian countries, aligned with research questions 2, 3, and 4, by adding the terms (“South Asia” OR India OR Bangladesh OR Nepal OR Bhutan OR “Sri Lanka” OR Pakistan OR Maldives) to Phase 1. This phase identified a total of 123 studies.

In Phase 3, search 2 was further narrowed in search 3A by adding the study type filters to answer RQ 2, which yielded 104 results, followed by search 3B, which involved adding the key terms (prevalence OR epidemiology OR incidence OR demography) AND (factors OR determinants OR etiology OR “socio-demographic”) to answer RQ 4; this search identified 64 studies. Finally, in phase 4, search 3A was further refined in search 4 to answer RQ 3 (to extract the evidence on interventions aimed at addressing maternal micronutrient deficiencies and their effectiveness on infant neurodevelopment outcomes) as described in Figure 1, after which a total of 81 studies were obtained.

In Step 2, the PubMed search string will be adapted and applied to the following:

- Electronic databases: PubMed, MEDLINE, Embase, Google Scholar, Cochrane Library, Open Gray, JSTOR, Wiley.
- Trial registers (eg, ClinicalTrials.gov, PROSPERO [International Prospective Register of Systematic Reviews])
- Manual searches of key journals (we will identify common journal titles like *BMC Pregnancy and Childbirth*, *The Journal of Nutrition*, *The American Journal of Clinical Nutrition*, *BMC Public Health*, *Public Health Nutrition*, *BMC Nutrition*, *Maternal and Child Nutrition,* and *Nutrients*)

The history of electronic database searches will be documented in Multimedia Appendix 2.

As a third step, the snowballing method will be used to examine the bibliographies of identified initial articles to find related and potentially relevant papers to again set the starting point. Both backward (reference tracking) and forward (citation tracking) snowballing methods will be used. As presented in the selection criteria, only studies published in English will be considered. The search will not be restricted by publication year and all studies published prior to June 2025 will be included. This inclusive time frame is intended to capture all the available evidence on maternal micronutrient status and its association with the neurodevelopment of offspring. Duplicate studies will be removed using the Rayyan software [25].

#### Information Sources

This review will include both primary and secondary research with the following range of methodologies: experimental studies (randomized controlled trials, nonrandomized controlled trials) on the association of maternal micronutrients and neurodevelopment of offspring; observational studies (prospective and retrospective cohort studies, case-control studies, analytical cross-sectional studies); systematic, scoping, and narrative reviews; qualitative studies; and mixed method studies.

### Stage 3: Study Selection

#### Eligibility Criteria

The eligibility criteria for study selection were also formulated using the PCC framework and are elaborated in Table 1.

**Table 1.** Eligibility criteria based on Population, Concept, and Context.

Category	Inclusion criteria	Exclusion criteria
Population	Studies that measured micronutrient status during pregnancy (15‐49 y) AND reported neurodevelopment of their infants up to 2 years of age AND/OR Studies that administered single/multiple micronutrient(s) during pregnancy with or without infant supplementation, by any modality (oral/parenteral), and assessed the neurodevelopment of infants using a standard scale AND Studies conducted on human subjects AND Studies published in English AND All studies published prior to July 2025	Studies conducted on women with multiple pregnancy OR Studies conducted on pregnant women with known or prediagnosed chronic medical conditions, mental health disorders, substance abuse, or infertility treatment OR Studies conducted on infants born extremely preterm, severe small for gestational age, or with neonatal neural disorders OR Non-English articles
Concept	Gestation is defined as the period between conception and birth. Micronutrients will include all vitamins and minerals required by the body in very small amounts. Neurodevelopmental domains will include motor, language, cognitive/problem-solving using a standardized scale of assessment like the Bayley Scales of Infant and Toddler Development, Standardized Infant NeuroDevelopmental Assessment, or Developmental Assessment Scales for Indian Infants Infancy will be defined as the period up to 2 years South Asian countries: specifically, India, Bhutan, Bangladesh, Nepal, and Sri Lanka	—^a^
Context	Studies conducted to establish the relationship between maternal micronutrient status during pregnancy and neurodevelopment of offspring during infancy	—^a^

a Not applicable.

Study selection will be made by two independent reviewers using the study selection form. Separate study selection forms have been developed and standardized for screening at two levels (Multimedia Appendix 3). For standardization, the reviewers’ agreement was measured using the Cohen κ statistic of 0.8 or higher for every question (title and abstract screening: 0.96 and full-text screening: 1) [26,27]. For the title and abstract screening form, the first 50 studies from search results after randomization were screened. Ten studies identified for inclusion through title and abstract screening were screened for full text. The forms were modified based on discussions to resolve reviewers’ disagreements. Study selection will be made at two levels:

- Level 1: Title and abstract screening. After the removal of duplicates, all the abstracts retrieved from the search will be screened using the title and abstract screening form. Each study will be categorized as “YES,” “NO,” or “UNCLEAR.” Studies marked as “Yes” or “Unclear” will be included for full-text review. Reasons for exclusion will be documented for the excluded studies to ensure transparency.
- Level 2: Full-text review. All studies marked as “YES” during level 1 screening will be retrieved in full for this level. Two independent reviewers will screen all the full-text articles using the final eligibility for inclusion in the scoping review. Studies that best fit with the research question will be included, while others will be excluded for documented reasons. Any discordant full-text articles will be reviewed for a second time, and further disagreement about study eligibility at this level will be resolved through discussion with a third reviewer until full consensus is obtained. The unavailability of a full-text article will also be documented. An attempt will be made to contact the authors to request the full text or clarify any concerns.

The references will be downloaded and then imported into a reference library using Zotero (version 7.0.13, 64-bit; Corporation for Digital Scholarship). The PRISMA flow diagram for scoping reviews [28] will be used to graphically depict the movement of sources through the search process to eventual inclusion. A narrative description of the selection process for included sources of evidence will be provided, based on the inclusion and exclusion criteria.

#### Data Variables

Data will be collected on the following variables:

- Primary outcomes: Direct measures of neurodevelopment in infants up to 2 years of age. These will include development quotients of cognitive development, motor development (both gross and fine), language acquisition, and social-emotional development.
- Secondary outcomes: Maternal micronutrient levels, birth outcomes, infant growth, and biochemical indicators.
- Determinants: Additional factors such as the type, duration, and timing of micronutrient supplementation; the timing of neurodevelopmental assessments; and socioeconomic or demographic correlates that may influence neurodevelopmental outcomes.
- Safety profile: Data related to the safety of maternal micronutrient exposure during pregnancy will also be extracted. This includes maternal adverse effects such as symptoms of toxicity or gastrointestinal disturbances, as well as infant-related adverse outcomes resulting from micronutrient excesses or deficiencies.

### Stage 4: Data Collection

The next stage will be “charting” of key information derived from the included studies, which will also be an iterative process. According to Arksey and O’Malley’s framework [21], charting is described as a technique for synthesizing and interpreting qualitative data by sifting, charting, and sorting material according to key issues and themes. Two structured data-charting forms have been developed around the PCC framework [29] as per RQ 1 based on a global basis and for subsequent research questions based on South Asian countries. The data-charting forms were developed and refined after pilot-testing by two independent reviewers on the first 5 studies meeting inclusion criteria to enhance clarity and comprehensiveness (Multimedia Appendix 4). Discrepancies or inconsistencies in data extraction were resolved through discussion between the reviewers. If disagreements persisted, a third reviewer was consulted to mediate and finalize the data extraction form. The extracted data will be compiled into a single Microsoft Excel spreadsheet for validation and coding.

JBI’s quality appraisal tools will be used [30] for quality appraisal of studies identified after full-text screening for RQ 3.

### Stage 5: Data Summary and Synthesis of Results

Data extraction will be conducted independently by two reviewers using standardized forms anchored in the PCC framework, with iterative refinement following pilot-testing. Quantitative variables will include study design, setting and country, sampling frame, gestational timing of micronutrient measurement or supplementation, definition and assay methods for micronutrient status, infant age at assessment (≤24 mo), neurodevelopmental instruments used (eg, Bayley Scales of Infant and Toddler Development, Standardized Infant NeuroDevelopmental Assessment, Developmental Assessment Scales for Indian Infants), and outcome domains (cognitive, language, motor, social-emotional). Descriptive statistics (frequencies, percentages, means, ranges) will summarize study features and outcome distributions. Results will be presented in structured tables and charts to directly address RQs 1‐4 (RQ 1: which deficiencies are associated with infant neurodevelopment; RQ 2: quality/spectrum of evidence; RQ 3: interventions and effectiveness; RQ 4: prevalence and sociodemographic determinants). Qualitative evidence will be analyzed inductively using ATLAS.ti with open coding to identify concepts related to pathways, measurement heterogeneity, contextual determinants, and implementation barriers and enablers. Codes will be grouped into categories and higher-order themes will be aligned to the review objectives and RQs. Integration will proceed via a convergent narrative synthesis: quantitative summaries will establish the breadth and patterning of exposures and outcomes, while qualitative themes will provide interpretive depth around mechanisms and context. The synthesis will explicitly map findings to each review question. For studies pertinent to RQ 3 (interventions), methodological quality will be assessed using the relevant JBI critical appraisal checklists; quality judgments will be reported in an appraisal table and used to qualify interpretations. All stages of conduct and reporting will adhere to the PRISMA-ScR checklist (Checklist 1) to ensure transparency and reproducibility.

### Stage 6: Consultation

Stakeholders, including health care professionals, researchers, and policymakers, will be consulted to provide insights and feedback on the review’s findings. The consultation exercise will help refine the review’s scope, identify additional relevant studies, and ensure the findings are applicable to real-world settings.

## Results

This scoping review was funded in January 2025 and formally began in February 2025. The final scoping review will be performed and reported according to this protocol and the PRISMA-ScR guidelines. On July 3, 2025, the initial search was conducted on PubMed followed by other databases. Initially, the search returned 12,741 records, and 10,992 records were screened by title and abstract for eligibility after removing duplicate records. A total of 283 studies were identified by abstract screening; as of July 2025, full-text screening is in progress to determine their eligibility. The final synthesis, including a completed PRISMA-ScR flow diagram with study counts at each stage, is anticipated by January 2026.

## Discussion

This scoping review is expected to fill an important void by systematically collating and summarizing the available evidence on maternal micronutrient deficiencies and infant neurodevelopment in South Asia. By charting a range of studies—including observational cohorts, trials, and reviews—it will assess the breadth of evidence and highlight patterns or inconsistencies in findings across the region. In doing so, it is expected to highlight research gaps (for example, a lack of long-term neurodevelopmental follow-up studies or an underrepresentation of certain regions or micronutrients or a poor understanding of sociodemographic influences) and suggest priorities for deeper investigation (systematic reviews and meta-analyses). The review will document the prevalence and distribution of key micronutrient deficiencies in South Asian countries and their associated sociodemographic determinants, providing valuable epidemiological context for policymakers. The findings, when integrated with those from a concurrent policy review of South Asian countries, will provide valuable insights into the most prevalent deficiencies and the populations most affected. This understanding can enhance the effectiveness of targeted interventions.

Compared to previous global work [7], this scoping review offers a more focused synthesis of evidence specific to maternal micronutrients, South Asia, and neurodevelopment, addressing a notable gap in the literature. Although earlier global reviews have explored the relationship between maternal nutrition and infant neurodevelopment, few have systematically mapped the prevalence, determinants, and intervention landscape for micronutrient deficiencies in this region. By including a broad range of study designs and emphasizing regional context, this review provides a nuanced understanding that complements and extends prior work.

A key strength of this review is its comprehensive and systematic methodology, which adheres to an established framework (JBI) and aligns with PRISMA-ScR guidelines. The inclusion of multiple study designs and gray literature extends the comprehensiveness of the analysis beyond trials published in indexed journals. By focusing specifically on the South Asian context, the review allows for a more nuanced understanding of regional contexts. Furthermore, integrating findings with concurrent qualitative assessments will enhance interpretability. Nevertheless, several limitations are recognized. The scope of the review is restricted to studies examining maternal micronutrient deficiencies exclusively in relation to neurodevelopment, which may affect the generalizability of the results. The review’s limitation to English-language publications introduces the potential for language bias. Variability among study designs, definitions of micronutrient deficiency, and neurodevelopmental assessment tools may hinder comparability and limit the feasibility of quantitative synthesis. Additionally, publication bias may obscure null or negative findings.

Findings will guide subsequent collaborative project activities, including feasibility assessments, biomarker refinement, and intervention design. They will also guide the development of a comprehensive research agenda focusing on underexplored micronutrients, long-term outcomes, and integrated maternal-infant nutrition interventions. This scoping review will also have important implications for both research and policy. For researchers, a synthesized overview of existing studies will illuminate what is known [7] and unknown, thereby informing the design of future studies such as trials of multiple micronutrient interventions or long-term longitudinal studies tracking developmental outcomes or evaluating the dimensions of neurodevelopment impacted by single micronutrient deficiencies. For policymakers and public health authorities in South Asia, the review’s findings can guide nutritional strategies to improve early childhood development. If the evidence establishes, for example, that maternal deficiencies in nutrients like vitamin B12 or vitamin D are linked to developmental delays, antenatal care programs could broaden their supplementation schemes beyond iron and folate or implement screening for these deficiencies in high-risk groups. Several South Asian countries are already considering a transition from iron and folic acid supplements to multiple micronutrient supplements in pregnancy, in line with updated global guidelines, to better address the spectrum of deficiencies affecting mothers and infants [4,20]. Evidence from this scoping review could support such policy shifts by providing region-specific data on which micronutrient interventions yield meaningful benefits for child development. Ultimately, by elucidating the relationships between maternal micronutrient status and infant neurodevelopment in South Asia, this work will contribute to shaping more effective nutritional policies and research agendas aimed at ensuring children in this populous region achieve their full developmental potential.

Results will be disseminated through peer-reviewed publications, conference presentations, social media, and stakeholder workshops convened across partner countries. Policy briefs and visual summaries will target public health officials, program managers, nongovernmental organizations, and frontline providers to support translation into antenatal nutrition programs and early childhood development strategies.

## Supplementary material

10.2196/81592 Multimedia Appendix 1 Search queries and details.

10.2196/81592 Multimedia Appendix 2 Draft table for the electronic database search history.

10.2196/81592 Multimedia Appendix 3 Screening forms.

10.2196/81592 Multimedia Appendix 4 Data extraction form.

10.2196/81592 Checklist 1 PRISMA checklist.

10.2196/81592 Peer Review Report 1 Peer review report by the National Institute for Health Research (NIHR) Global Health Research Development Awards Review Committee, Department of Health and Social Care (United Kingdom).

## Abbreviations

JBI: Joanna Briggs Institute
PCC: Population, Concept, Context
PRISMA-ScR: Preferred Reporting Items for Systematic Reviews and Meta-Analyses extension for Scoping Reviews
PROSPERO: International Prospective Register of Systematic Reviews
RQ: research question
SACMIND: South Asia Collaborative for Maternal Micronutrients and Infant Neurodevelopment
