# Supplementary material for: Maternal Micronutrient Status During Pregnancy and Its Neurodevelopmental Implications for Infants in South Asia: Protocol for a Scoping Review
Source: JMIR Res Protoc. 2025 Dec 15;14:e81592. doi: 10.2196/81592 (PMC12705126; doi:10.2196/81592)
Supplement: Multimedia Appendix 1 [file resprot-v14-e81592-s001.pdf]

Appendix 1

Search	Query	Search Details	Results retrieved
Search 1	((pregnancy) OR (maternal)) AND ((infant) OR (offspring) OR (child)) AND ((micronutrient status) OR (micronutrient deficiency) OR (vitamin A) OR (vitamin B) OR (vitamin C) OR (vitamin D) OR (vitamin E) OR (vitamin K) OR (thiamin) OR (riboflavin) OR (niacin) OR (B5) OR (B6) OR (biotin) OR (folic acid) OR (cobalmin) OR (Iron) OR (calcium) OR (sulfur) OR (magnesium) OR (phosphorus) OR (sodium) OR (potassium) OR (zinc) OR (copper) OR (manganese) OR (molybdenum) OR (boron) OR	(((((("pregnancy"[MeSH Terms] OR "pregnancy"[All Fields] OR "pregnancies"[All Fields] OR "pregnancy s"[All Fields] OR ("maternally"[All Fields] OR "maternities"[All Fields] OR "maternity"[All Fields] OR "mothers"[MeSH Terms] OR "mothers"[All Fields] OR "maternal"[All Fields])) AND ("infant"[MeSH Terms] OR "infant"[All Fields] OR "infants"[All Fields] OR "infant s"[All Fields] OR ("offspring"[All Fields] OR "offspring s"[All Fields] OR "offsprings"[All Fields]) OR ("child"[MeSH Terms] OR "child"[All Fields] OR "children"[All Fields] OR "child s"[All Fields] OR "children s"[All Fields] OR "childrens"[All Fields] OR "childs"[All Fields])) AND ((("micronutrients"[Pharmacological Action] OR "micronutrients"[Supplementary Concept] OR "micronutrients"[All Fields] OR "micronutrients"[MeSH Terms] OR "micronutriments"[All Fields] OR "trace elements"[Pharmacological Action] OR "trace elements"[Supplementary Concept] OR "trace elements"[All Fields] OR "micronutrient"[All Fields] OR "trace elements"[MeSH Terms] OR ("trace"[All Fields] AND "elements"[All Fields])) AND "status"[All Fields]) OR (("micronutrients"[Pharmacological Action] OR "micronutrients"[Supplementary Concept] OR "micronutrients"[All Fields] OR "micronutrients"[MeSH Terms] OR "micronutriments"[All Fields] OR "trace elements"[Pharmacological Action] OR "trace elements"[Supplementary Concept] OR "trace elements"[All Fields] OR "micronutrient"[All Fields] OR "trace elements"[MeSH Terms] OR ("trace"[All Fields] AND "elements"[All Fields])) AND ("deficiences"[All Fields] OR "deficiencies"[All Fields] OR "deficiency"[MeSH Subheading] OR "deficiency"[All Fields] OR "deficient"[All Fields] OR "deficients"[All Fields])) OR ("vitamin a"[Supplementary Concept] OR "vitamin a"[All Fields] OR "vitamin a"[MeSH Terms]) OR ("vitamin b complex"[Pharmacological Action] OR "vitamin b complex"[Supplementary Concept] OR "vitamin b complex"[All Fields] OR "vitamin b"[All Fields] OR "vitamin b complex"[MeSH Terms]) OR ("ascorbic acid"[Supplementary Concept] OR "ascorbic acid"[All Fields] OR "vitamin c"[All Fields] OR "ascorbic acid"[MeSH Terms] OR ("ascorbic"[All Fields] AND "acid"[All Fields])) OR ("vitamin d"[Supplementary Concept] OR "vitamin d"[All Fields] OR "ergocalciferols"[Supplementary Concept] OR "ergocalciferols"[All Fields] OR "vitamin d"[MeSH Terms] OR "ergocalciferols"[MeSH Terms]) OR ("vitamin e"[Supplementary Concept] OR "vitamin e"[All Fields] OR "vitamin e"[MeSH Terms]) OR ("vitamin k"[Supplementary Concept] OR "vitamin k"[All Fields] OR "vitamin k"[MeSH Terms]) OR ("thiamine"[Supplementary Concept] OR "thiamine"[All Fields] OR "thiamin"[All Fields] OR "thiamine"[MeSH Terms] OR "thiamines"[All Fields]) OR ("riboflavin"[Supplementary Concept] OR "riboflavin"[All Fields] OR "riboflavin"[MeSH Terms] OR "riboflavine"[All Fields] OR "riboflavins"[All Fields]) OR ("niacin"[Supplementary Concept] OR "niacin"[All Fields] OR "niacin"[MeSH Terms] OR	1172

	(chlorine) OR (chloride) OR (selenium) OR (cobalt) OR (fluorine) OR (fluoride) OR (iodine) OR (silicon)) AND ((neurodevelopment) OR ("brain development") OR ("motor development") OR ("psychomotor development") OR ("language development") OR ("cognitive development") OR (Cognition)) NOT (toxic) NOT (gene) NOT (autism) NOT (preterm)	"niacin s"[All Fields] OR "niacinate"[All Fields] OR "niacine"[All Fields] OR "niacins"[All Fields]) OR "B5"[All Fields] OR "B6"[All Fields] OR ("biotin"[Supplementary Concept] OR "biotin"[All Fields] OR "biotin"[MeSH Terms] OR "biotine"[All Fields] OR "biotinated"[All Fields] OR "biotins"[All Fields]) OR ("folic acid"[Supplementary Concept] OR "folic acid"[All Fields] OR "folic acid"[MeSH Terms] OR ("folic"[All Fields] AND "acid"[All Fields])) OR "cobalmin"[All Fields] OR ("iron"[Supplementary Concept] OR "iron"[All Fields] OR "iron"[MeSH Terms]) OR ("calcium"[Supplementary Concept] OR "calcium"[All Fields] OR "calcium"[MeSH Terms] OR "calciums"[All Fields] OR "calcium s"[All Fields]) OR ("sulfur"[Supplementary Concept] OR "sulfur"[All Fields] OR "sulfurs"[All Fields] OR "sulfur"[MeSH Terms] OR "sulfur s"[All Fields] OR "sulfurated"[All Fields] OR "sulfuration"[All Fields] OR "sulfuric"[All Fields] OR "sulfurization"[All Fields] OR "sulfurize"[All Fields] OR "sulfurized"[All Fields] OR "sulfurizing"[All Fields] OR "sulfurous"[All Fields] OR "sulphur"[All Fields] OR "sulphurated"[All Fields] OR "sulphuric"[All Fields] OR "sulphurous"[All Fields] OR "sulphurs"[All Fields]) OR ("magnesium"[Supplementary Concept] OR "magnesium"[All Fields] OR "magnesium"[MeSH Terms] OR "magnesium s"[All Fields] OR "magnesiums"[All Fields]) OR ("phosphorus"[Supplementary Concept] OR "phosphorus"[All Fields] OR "phosphorus"[MeSH Terms]) OR ("sodium"[Supplementary Concept] OR "sodium"[All Fields] OR "sodium"[MeSH Terms] OR "sodiums"[All Fields]) OR ("potassium dietary"[Supplementary Concept] OR "potassium dietary"[All Fields] OR "potassium"[All Fields] OR "potassium"[Supplementary Concept] OR "potassium, dietary"[MeSH Terms] OR ("potassium"[All Fields] AND "dietary"[All Fields]) OR "dietary potassium"[All Fields] OR "potassium"[MeSH Terms]) OR ("zinc"[Supplementary Concept] OR "zinc"[All Fields] OR "zinc"[MeSH Terms]) OR ("copper"[Supplementary Concept] OR "copper"[All Fields] OR "copper"[MeSH Terms] OR "coppers"[All Fields] OR "copper s"[All Fields]) OR ("manganese"[Supplementary Concept] OR "manganese"[All Fields] OR "manganese"[MeSH Terms] OR "manganeses"[All Fields]) OR ("molybdenum"[Supplementary Concept] OR "molybdenum"[All Fields] OR "molybdenum"[MeSH Terms]) OR ("boron"[Supplementary Concept] OR "boron"[All Fields] OR "boron"[MeSH Terms] OR "boron s"[All Fields] OR "boronate"[All Fields] OR "boronated"[All Fields] OR "boronates"[All Fields] OR "boronation"[All Fields] OR "boronic"[All Fields] OR "borons"[All Fields]) OR ("chlorin"[Supplementary Concept] OR "chlorin"[All Fields] OR "chlorinate"[All Fields] OR "chlorinated"[All Fields] OR "chlorinates"[All Fields] OR "chlorinating"[All Fields] OR "chlorinations"[All Fields] OR "chlorinator"[All Fields] OR "chlorinators"[All Fields] OR "chlorine"[Supplementary Concept] OR "chlorine"[All Fields] OR "chlorine"[MeSH Terms] OR "chlorine s"[All Fields] OR "chlorines"[All Fields] OR "chlorins"[All Fields] OR "halogenation"[MeSH Terms] OR "halogenation"[All Fields] OR "chlorination"[All Fields]) OR ("chlorid"[All Fields] OR "chlorides"[Supplementary Concept] OR "chlorides"[All Fields] OR "chloride"[All Fields] OR "chlorides"[MeSH Terms]) OR ("selenium"[Supplementary	

		Concept] OR "selenium"[All Fields] OR "selenium"[MeSH Terms] OR "selenium s"[All Fields] OR "seleniums"[All Fields]) OR ("cobalt"[Supplementary Concept] OR "cobalt"[All Fields] OR "cobalt"[MeSH Terms] OR "cobaltate"[All Fields] OR "cobaltates"[All Fields] OR "cobaltic"[All Fields] OR "cobaltous"[All Fields]) OR ("fluorinate"[All Fields] OR "fluorinated"[All Fields] OR "fluorinates"[All Fields] OR "fluorinating"[All Fields] OR "fluorinations"[All Fields] OR "fluorinative"[All Fields] OR "fluorine"[Supplementary Concept] OR "fluorine"[All Fields] OR "fluorine"[MeSH Terms] OR "fluorines"[All Fields] OR "fluorine s"[All Fields] OR "halogenation"[MeSH Terms] OR "halogenation"[All Fields] OR "fluorination"[All Fields]) OR ("fluoridate"[All Fields] OR "fluoridated"[All Fields] OR "fluoridating"[All Fields] OR "fluoridation"[MeSH Terms] OR "fluoridation"[All Fields] OR "fluoridation s"[All Fields] OR "fluoride s"[All Fields] OR "fluorided"[All Fields] OR "fluorides"[Supplementary Concept] OR "fluorides"[All Fields] OR "fluoride"[All Fields] OR "fluorides"[MeSH Terms] OR "fluoridization"[All Fields] OR "fluoridized"[All Fields]) OR ("halogenation"[MeSH Terms] OR "halogenation"[All Fields] OR "iodination"[All Fields] OR "iodin"[All Fields] OR "iodinate"[All Fields] OR "iodinated"[All Fields] OR "iodinates"[All Fields] OR "iodinating"[All Fields] OR "iodinations"[All Fields] OR "iodine"[Supplementary Concept] OR "iodine"[All Fields] OR "iodine"[MeSH Terms] OR "iodines"[All Fields]) OR ("silicon"[Supplementary Concept] OR "silicon"[All Fields] OR "silicon"[MeSH Terms] OR "silicon s"[All Fields] OR "silicons"[All Fields])) AND ("neurodevelopment"[All Fields] OR "brain development"[All Fields] OR "motor development"[All Fields] OR "psychomotor development"[All Fields] OR "language development"[All Fields] OR "cognitive development"[All Fields] OR ("cognition"[MeSH Terms] OR "cognition"[All Fields] OR "cognitions"[All Fields] OR "cognitive"[All Fields] OR "cognitively"[All Fields] OR "cognitives"[All Fields]))) NOT ("toxic"[All Fields] OR "toxical"[All Fields] OR "toxically"[All Fields] OR "toxicant"[All Fields] OR "toxicant s"[All Fields] OR "toxicants"[All Fields] OR "toxicated"[All Fields] OR "toxication"[All Fields] OR "toxicities"[All Fields] OR "toxicity"[MeSH Subheading] OR "toxicity"[All Fields] OR "toxicity s"[All Fields] OR "toxics"[All Fields])) NOT ("genes"[MeSH Terms] OR "genes"[All Fields] OR "gene"[All Fields])) NOT ("autism s"[All Fields] OR "autisms"[All Fields] OR "autistic disorder"[MeSH Terms] OR ("autistic"[All Fields] AND "disorder"[All Fields]) OR "autistic disorder"[All Fields] OR "autism"[All Fields])) NOT ("preterm"[All Fields] OR "preterms"[All Fields])) AND ((humans[Filter]) AND (english[Filter]))	
**Search 2**	AND ("South Asia" OR India OR Bangladesh OR Nepal OR Bhutan OR "Sri Lanka" OR	((((((("pregnancy"[MeSH Terms] OR "pregnancy"[All Fields] OR "pregnancies"[All Fields] OR "pregnancy s"[All Fields] OR ("maternally"[All Fields] OR "maternities"[All Fields] OR "maternity"[All Fields] OR "mothers"[MeSH Terms] OR "mothers"[All Fields] OR "maternal"[All Fields])) AND ("infant"[MeSH Terms] OR "infant"[All Fields] OR "infants"[All Fields] OR "infant s"[All Fields] OR ("offspring"[All Fields] OR "offspring s"[All Fields] OR "offsprings"[All Fields]) OR ("child"[MeSH Terms] OR "child"[All Fields] OR "children"[All Fields] OR "child s"[All Fields] OR "children s"[All Fields] OR	124

	Pakistan OR Maldives)	"childrens"[All Fields] OR "childs"[All Fields])) AND ((("micronutrients"[Pharmacological Action] OR "micronutrients"[Supplementary Concept] OR "micronutrients"[All Fields] OR "micronutrients"[MeSH Terms] OR "micronutriments"[All Fields] OR "trace elements"[Pharmacological Action] OR "trace elements"[Supplementary Concept] OR "trace elements"[All Fields] OR "micronutrient"[All Fields] OR "trace elements"[MeSH Terms] OR ("trace"[All Fields] AND "elements"[All Fields])) AND "status"[All Fields]) OR (("micronutrients"[Pharmacological Action] OR "micronutrients"[Supplementary Concept] OR "micronutrients"[All Fields] OR "micronutrients"[MeSH Terms] OR "micronutriments"[All Fields] OR "trace elements"[Pharmacological Action] OR "trace elements"[Supplementary Concept] OR "trace elements"[All Fields] OR "micronutrient"[All Fields] OR "trace elements"[MeSH Terms] OR ("trace"[All Fields] AND "elements"[All Fields])) AND ("deficiences"[All Fields] OR "deficiencies"[All Fields] OR "deficiency"[MeSH Subheading] OR "deficiency"[All Fields] OR "deficient"[All Fields] OR "deficients"[All Fields])) OR ("vitamin a"[Supplementary Concept] OR "vitamin a"[All Fields] OR "vitamin a"[MeSH Terms]) OR ("vitamin b complex"[Pharmacological Action] OR "vitamin b complex"[Supplementary Concept] OR "vitamin b complex"[All Fields] OR "vitamin b"[All Fields] OR "vitamin b complex"[MeSH Terms]) OR ("ascorbic acid"[Supplementary Concept] OR "ascorbic acid"[All Fields] OR "vitamin c"[All Fields] OR "ascorbic acid"[MeSH Terms] OR ("ascorbic"[All Fields] AND "acid"[All Fields])) OR ("vitamin d"[Supplementary Concept] OR "vitamin d"[All Fields] OR "ergocalciferols"[Supplementary Concept] OR "ergocalciferols"[All Fields] OR "vitamin d"[MeSH Terms] OR "ergocalciferols"[MeSH Terms]) OR ("vitamin e"[Supplementary Concept] OR "vitamin e"[All Fields] OR "vitamin e"[MeSH Terms]) OR ("vitamin k"[Supplementary Concept] OR "vitamin k"[All Fields] OR "vitamin k"[MeSH Terms]) OR ("thiamine"[Supplementary Concept] OR "thiamine"[All Fields] OR "thiamin"[All Fields] OR "thiamine"[MeSH Terms] OR "thiamines"[All Fields]) OR ("riboflavin"[Supplementary Concept] OR "riboflavin"[All Fields] OR "riboflavin"[MeSH Terms] OR "riboflavine"[All Fields] OR "riboflavins"[All Fields]) OR ("niacin"[Supplementary Concept] OR "niacin"[All Fields] OR "niacin"[MeSH Terms] OR "niacin s"[All Fields] OR "niacinate"[All Fields] OR "niacine"[All Fields] OR "niacins"[All Fields]) OR "B5"[All Fields] OR "B6"[All Fields] OR ("biotin"[Supplementary Concept] OR "biotin"[All Fields] OR "biotin"[MeSH Terms] OR "biotine"[All Fields] OR "biotinated"[All Fields] OR "biotins"[All Fields]) OR ("folic acid"[Supplementary Concept] OR "folic acid"[All Fields] OR "folic acid"[MeSH Terms] OR ("folic"[All Fields] AND "acid"[All Fields])) OR "cobalmin"[All Fields] OR ("iron"[Supplementary Concept] OR "iron"[All Fields] OR "iron"[MeSH Terms]) OR ("calcium"[Supplementary Concept] OR "calcium"[All Fields] OR "calcium"[MeSH Terms] OR "calciums"[All Fields] OR "calcium s"[All Fields]) OR ("sulfur"[Supplementary Concept] OR "sulfur"[All Fields] OR "sulfurs"[All Fields] OR "sulfur"[MeSH Terms] OR "sulfur s"[All Fields] OR "sulfurated"[All Fields] OR "sulfuration"[All Fields] OR "sulfuric"[All Fields] OR	

		"sulfurization"[All Fields] OR "sulfurize"[All Fields] OR "sulfurized"[All Fields] OR "sulfurizing"[All Fields] OR "sulfurous"[All Fields] OR "sulphur"[All Fields] OR "sulphurated"[All Fields] OR "sulphuric"[All Fields] OR "sulphurous"[All Fields] OR "sulphurs"[All Fields]) OR ("magnesium"[Supplementary Concept] OR "magnesium"[All Fields] OR "magnesium"[MeSH Terms] OR "magnesium s"[All Fields] OR "magnesiums"[All Fields]) OR ("phosphorus"[Supplementary Concept] OR "phosphorus"[All Fields] OR "phosphorus"[MeSH Terms]) OR ("sodium"[Supplementary Concept] OR "sodium"[All Fields] OR "sodium"[MeSH Terms] OR "sodiums"[All Fields]) OR ("potassium dietary"[Supplementary Concept] OR "potassium dietary"[All Fields] OR "potassium"[All Fields] OR "potassium"[Supplementary Concept] OR "potassium, dietary"[MeSH Terms] OR ("potassium"[All Fields] AND "dietary"[All Fields]) OR "dietary potassium"[All Fields] OR "potassium"[MeSH Terms]) OR ("zinc"[Supplementary Concept] OR "zinc"[All Fields] OR "zinc"[MeSH Terms]) OR ("copper"[Supplementary Concept] OR "copper"[All Fields] OR "copper"[MeSH Terms] OR "coppers"[All Fields] OR "copper s"[All Fields]) OR ("manganese"[Supplementary Concept] OR "manganese"[All Fields] OR "manganese"[MeSH Terms] OR "manganeses"[All Fields]) OR ("molybdenum"[Supplementary Concept] OR "molybdenum"[All Fields] OR "molybdenum"[MeSH Terms]) OR ("boron"[Supplementary Concept] OR "boron"[All Fields] OR "boron"[MeSH Terms] OR "boron s"[All Fields] OR "boronate"[All Fields] OR "boronated"[All Fields] OR "boronates"[All Fields] OR "boronation"[All Fields] OR "boronic"[All Fields] OR "borons"[All Fields]) OR ("chlorin"[Supplementary Concept] OR "chlorin"[All Fields] OR "chlorinate"[All Fields] OR "chlorinated"[All Fields] OR "chlorinates"[All Fields] OR "chlorinating"[All Fields] OR "chlorinations"[All Fields] OR "chlorinator"[All Fields] OR "chlorinators"[All Fields] OR "chlorine"[Supplementary Concept] OR "chlorine"[All Fields] OR "chlorine"[MeSH Terms] OR "chlorine s"[All Fields] OR "chlorines"[All Fields] OR "chlorins"[All Fields] OR "halogenation"[MeSH Terms] OR "halogenation"[All Fields] OR "chlorination"[All Fields]) OR ("chlorid"[All Fields] OR "chlorides"[Supplementary Concept] OR "chlorides"[All Fields] OR "chloride"[All Fields] OR "chlorides"[MeSH Terms]) OR ("selenium"[Supplementary Concept] OR "selenium"[All Fields] OR "selenium"[MeSH Terms] OR "selenium s"[All Fields] OR "seleniums"[All Fields]) OR ("cobalt"[Supplementary Concept] OR "cobalt"[All Fields] OR "cobalt"[MeSH Terms] OR "cobaltate"[All Fields] OR "cobaltates"[All Fields] OR "cobaltic"[All Fields] OR "cobaltous"[All Fields]) OR ("fluorinate"[All Fields] OR "fluorinated"[All Fields] OR "fluorinates"[All Fields] OR "fluorinating"[All Fields] OR "fluorinations"[All Fields] OR "fluorinative"[All Fields] OR "fluorine"[Supplementary Concept] OR "fluorine"[All Fields] OR "fluorine"[MeSH Terms] OR "fluorines"[All Fields] OR "fluorine s"[All Fields] OR "halogenation"[MeSH Terms] OR "halogenation"[All Fields] OR "fluorination"[All Fields]) OR ("fluoridate"[All Fields] OR "fluoridated"[All Fields] OR "fluoridating"[All Fields] OR "fluoridation"[MeSH Terms] OR "fluoridation"[All Fields] OR "fluoridation s"[All Fields] OR "fluoride s"[All Fields] OR	

		"fluorided"[All Fields] OR "fluorides"[Supplementary Concept] OR "fluorides"[All Fields] OR "fluoride"[All Fields] OR "fluorides"[MeSH Terms] OR "fluoridization"[All Fields] OR "fluoridized"[All Fields]) OR ("halogenation"[MeSH Terms] OR "halogenation"[All Fields] OR "iodination"[All Fields] OR "iodin"[All Fields] OR "iodinate"[All Fields] OR "iodinated"[All Fields] OR "iodinates"[All Fields] OR "iodinating"[All Fields] OR "iodinations"[All Fields] OR "iodine"[Supplementary Concept] OR "iodine"[All Fields] OR "iodine"[MeSH Terms] OR "iodines"[All Fields]) OR ("silicon"[Supplementary Concept] OR "silicon"[All Fields] OR "silicon"[MeSH Terms] OR "silicon s"[All Fields] OR "silicons"[All Fields])) AND ("neurodevelopment"[All Fields] OR "brain development"[All Fields] OR "motor development"[All Fields] OR "psychomotor development"[All Fields] OR "language development"[All Fields] OR "cognitive development"[All Fields] OR ("cognition"[MeSH Terms] OR "cognition"[All Fields] OR "cognitions"[All Fields] OR "cognitive"[All Fields] OR "cognitively"[All Fields] OR "cognitives"[All Fields]))) NOT ("toxic"[All Fields] OR "toxical"[All Fields] OR "toxically"[All Fields] OR "toxicant"[All Fields] OR "toxicant s"[All Fields] OR "toxicants"[All Fields] OR "toxicated"[All Fields] OR "toxication"[All Fields] OR "toxicities"[All Fields] OR "toxicity"[MeSH Subheading] OR "toxicity"[All Fields] OR "toxicity s"[All Fields] OR "toxics"[All Fields])) NOT ("genes"[MeSH Terms] OR "genes"[All Fields] OR "gene"[All Fields])) NOT ("autism s"[All Fields] OR "autisms"[All Fields] OR "autistic disorder"[MeSH Terms] OR ("autistic"[All Fields] AND "disorder"[All Fields]) OR "autistic disorder"[All Fields] OR "autism"[All Fields])) NOT ("preterm"[All Fields] OR "preterms"[All Fields])) AND ("South Asia"[All Fields] OR ("india"[MeSH Terms] OR "india"[All Fields] OR "india s"[All Fields] OR "indias"[All Fields]) OR ("bangladesh"[MeSH Terms] OR "bangladesh"[All Fields] OR "bangladesh s"[All Fields]) OR ("nepal"[MeSH Terms] OR "nepal"[All Fields] OR "nepal s"[All Fields]) OR ("bhutan"[MeSH Terms] OR "bhutan"[All Fields] OR "bhutan s"[All Fields]) OR "Sri Lanka"[All Fields] OR ("pakistan"[MeSH Terms] OR "pakistan"[All Fields] OR "pakistan s"[All Fields]) OR ("maldive"[All Fields] OR "maldives"[MeSH Terms] OR "maldives"[All Fields]))) AND ((humans[Filter]) AND (english[Filter]))	
Search 3 A	Adaptive Clinical Trial, Clinical Trial, Clinical Trial, Phase I, Clinical Trial, Phase II, Clinical Trial, Phase III, Clinical Trial, Phase IV, Clinical Trial	((((((("pregnancy"[MeSH Terms] OR "pregnancy"[All Fields] OR "pregnancies"[All Fields] OR "pregnancy s"[All Fields] OR ("maternally"[All Fields] OR "maternities"[All Fields] OR "maternity"[All Fields] OR "mothers"[MeSH Terms] OR "mothers"[All Fields] OR "maternal"[All Fields])) AND ("infant"[MeSH Terms] OR "infant"[All Fields] OR "infants"[All Fields] OR "infant s"[All Fields] OR ("offspring"[All Fields] OR "offspring s"[All Fields] OR "offsprings"[All Fields]) OR ("child"[MeSH Terms] OR "child"[All Fields] OR "children"[All Fields] OR "child s"[All Fields] OR "children s"[All Fields] OR "childrens"[All Fields] OR "childs"[All Fields])) AND ((("micronutrients"[Pharmacological Action] OR "micronutrients"[Supplementary Concept] OR "micronutrients"[All Fields] OR "micronutrients"[MeSH Terms] OR "micronutriments"[All Fields] OR "trace elements"[Pharmacological Action] OR "trace elements"[Supplementary Concept] OR	104

	Protocol, Comparative Study, Controlled Clinical Trial, English Abstract, Evaluation Study, Meta-Analysis, Multicenter Study, Network Meta-Analysis, Observational Study, Pragmatic Clinical Trial, Preprint, Randomized Controlled Trial, Research Support, N.I.H., Extramural, Research Support, N.I.H., Intramural, Research Support, Non-U.S. Gov't, Research Support, U.S. Gov't, Research Support, U.S. Gov't, Non-P.H.S., Research Support, U.S. Gov't, P.H.S., Retracted Publication, Retraction of	"trace elements"[All Fields] OR "micronutrient"[All Fields] OR "trace elements"[MeSH Terms] OR ("trace"[All Fields] AND "elements"[All Fields])) AND "status"[All Fields]) OR (("micronutrients"[Pharmacological Action] OR "micronutrients"[Supplementary Concept] OR "micronutrients"[All Fields] OR "micronutrients"[MeSH Terms] OR "micronutriments"[All Fields] OR "trace elements"[Pharmacological Action] OR "trace elements"[Supplementary Concept] OR "trace elements"[All Fields] OR "micronutrient"[All Fields] OR "trace elements"[MeSH Terms] OR ("trace"[All Fields] AND "elements"[All Fields])) AND ("deficiences"[All Fields] OR "deficiencies"[All Fields] OR "deficiency"[MeSH Subheading] OR "deficiency"[All Fields] OR "deficient"[All Fields] OR "deficients"[All Fields])) OR ("vitamin a"[Supplementary Concept] OR "vitamin a"[All Fields] OR "vitamin a"[MeSH Terms]) OR ("vitamin b complex"[Pharmacological Action] OR "vitamin b complex"[Supplementary Concept] OR "vitamin b complex"[All Fields] OR "vitamin b"[All Fields] OR "vitamin b complex"[MeSH Terms]) OR ("ascorbic acid"[Supplementary Concept] OR "ascorbic acid"[All Fields] OR "vitamin c"[All Fields] OR "ascorbic acid"[MeSH Terms] OR ("ascorbic"[All Fields] AND "acid"[All Fields])) OR ("vitamin d"[Supplementary Concept] OR "vitamin d"[All Fields] OR "ergocalciferols"[Supplementary Concept] OR "ergocalciferols"[All Fields] OR "vitamin d"[MeSH Terms] OR "ergocalciferols"[MeSH Terms]) OR ("vitamin e"[Supplementary Concept] OR "vitamin e"[All Fields] OR "vitamin e"[MeSH Terms]) OR ("vitamin k"[Supplementary Concept] OR "vitamin k"[All Fields] OR "vitamin k"[MeSH Terms]) OR ("thiamine"[Supplementary Concept] OR "thiamine"[All Fields] OR "thiamin"[All Fields] OR "thiamine"[MeSH Terms] OR "thiamines"[All Fields]) OR ("riboflavin"[Supplementary Concept] OR "riboflavin"[All Fields] OR "riboflavin"[MeSH Terms] OR "riboflavine"[All Fields] OR "riboflavins"[All Fields]) OR ("niacin"[Supplementary Concept] OR "niacin"[All Fields] OR "niacin"[MeSH Terms] OR "niacin s"[All Fields] OR "niacinate"[All Fields] OR "niacine"[All Fields] OR "niacins"[All Fields]) OR "B5"[All Fields] OR "B6"[All Fields] OR ("biotin"[Supplementary Concept] OR "biotin"[All Fields] OR "biotin"[MeSH Terms] OR "biotine"[All Fields] OR "biotinated"[All Fields] OR "biotins"[All Fields]) OR ("folic acid"[Supplementary Concept] OR "folic acid"[All Fields] OR "folic acid"[MeSH Terms] OR ("folic"[All Fields] AND "acid"[All Fields])) OR "cobalmin"[All Fields] OR ("iron"[Supplementary Concept] OR "iron"[All Fields] OR "iron"[MeSH Terms]) OR ("calcium"[Supplementary Concept] OR "calcium"[All Fields] OR "calcium"[MeSH Terms] OR "calciums"[All Fields] OR "calcium s"[All Fields]) OR ("sulfur"[Supplementary Concept] OR "sulfur"[All Fields] OR "sulfurs"[All Fields] OR "sulfur"[MeSH Terms] OR "sulfur s"[All Fields] OR "sulfurated"[All Fields] OR "sulfuration"[All Fields] OR "sulfuric"[All Fields] OR "sulfurization"[All Fields] OR "sulfurize"[All Fields] OR "sulfurized"[All Fields] OR "sulfurizing"[All Fields] OR "sulfurous"[All Fields] OR "sulphur"[All Fields] OR "sulphurated"[All Fields] OR "sulphuric"[All Fields] OR "sulphurous"[All Fields] OR "sulphurs"[All Fields]) OR ("magnesium"[Supplementary Concept] OR "magnesium"[All	

	Publication, Review, Scientific Integrity Review, Scoping Review, Systematic Review, Technical Report, Twin Study, Validation Study, English, Humans	Fields] OR "magnesium"[MeSH Terms] OR "magnesium s"[All Fields] OR "magnesiums"[All Fields]) OR ("phosphorus"[Supplementary Concept] OR "phosphorus"[All Fields] OR "phosphorus"[MeSH Terms]) OR ("sodium"[Supplementary Concept] OR "sodium"[All Fields] OR "sodium"[MeSH Terms] OR "sodiums"[All Fields]) OR ("potassium dietary"[Supplementary Concept] OR "potassium dietary"[All Fields] OR "potassium"[All Fields] OR "potassium"[Supplementary Concept] OR "potassium, dietary"[MeSH Terms] OR ("potassium"[All Fields] AND "dietary"[All Fields]) OR "dietary potassium"[All Fields] OR "potassium"[MeSH Terms]) OR ("zinc"[Supplementary Concept] OR "zinc"[All Fields] OR "zinc"[MeSH Terms]) OR ("copper"[Supplementary Concept] OR "copper"[All Fields] OR "copper"[MeSH Terms] OR "coppers"[All Fields] OR "copper s"[All Fields]) OR ("manganese"[Supplementary Concept] OR "manganese"[All Fields] OR "manganese"[MeSH Terms] OR "manganeses"[All Fields]) OR ("molybdenum"[Supplementary Concept] OR "molybdenum"[All Fields] OR "molybdenum"[MeSH Terms]) OR ("boron"[Supplementary Concept] OR "boron"[All Fields] OR "boron"[MeSH Terms] OR "boron s"[All Fields] OR "boronate"[All Fields] OR "boronated"[All Fields] OR "boronates"[All Fields] OR "boronation"[All Fields] OR "boronic"[All Fields] OR "borons"[All Fields]) OR ("chlorin"[Supplementary Concept] OR "chlorin"[All Fields] OR "chlorinate"[All Fields] OR "chlorinated"[All Fields] OR "chlorinates"[All Fields] OR "chlorinating"[All Fields] OR "chlorinations"[All Fields] OR "chlorinator"[All Fields] OR "chlorinators"[All Fields] OR "chlorine"[Supplementary Concept] OR "chlorine"[All Fields] OR "chlorine"[MeSH Terms] OR "chlorine s"[All Fields] OR "chlorines"[All Fields] OR "chlorins"[All Fields] OR "halogenation"[MeSH Terms] OR "halogenation"[All Fields] OR "chlorination"[All Fields]) OR ("chlorid"[All Fields] OR "chlorides"[Supplementary Concept] OR "chlorides"[All Fields] OR "chloride"[All Fields] OR "chlorides"[MeSH Terms]) OR ("selenium"[Supplementary Concept] OR "selenium"[All Fields] OR "selenium"[MeSH Terms] OR "selenium s"[All Fields] OR "seleniums"[All Fields]) OR ("cobalt"[Supplementary Concept] OR "cobalt"[All Fields] OR "cobalt"[MeSH Terms] OR "cobaltate"[All Fields] OR "cobaltates"[All Fields] OR "cobaltic"[All Fields] OR "cobaltous"[All Fields]) OR ("fluorinate"[All Fields] OR "fluorinated"[All Fields] OR "fluorinates"[All Fields] OR "fluorinating"[All Fields] OR "fluorinations"[All Fields] OR "fluorinative"[All Fields] OR "fluorine"[Supplementary Concept] OR "fluorine"[All Fields] OR "fluorine"[MeSH Terms] OR "fluorines"[All Fields] OR "fluorine s"[All Fields] OR "halogenation"[MeSH Terms] OR "halogenation"[All Fields] OR "fluorination"[All Fields]) OR ("fluoridate"[All Fields] OR "fluoridated"[All Fields] OR "fluoridating"[All Fields] OR "fluoridation"[MeSH Terms] OR "fluoridation"[All Fields] OR "fluoridation s"[All Fields] OR "fluoride s"[All Fields] OR "fluorided"[All Fields] OR "fluorides"[Supplementary Concept] OR "fluorides"[All Fields] OR "fluoride"[All Fields] OR "fluorides"[MeSH Terms] OR "fluoridization"[All Fields] OR "fluoridized"[All Fields]) OR ("halogenation"[MeSH Terms] OR "halogenation"[All Fields] OR "iodination"[All Fields] OR "iodin"[All Fields] OR "iodinate"[All Fields] OR	

		"iodinated"[All Fields] OR "iodinates"[All Fields] OR "iodinating"[All Fields] OR "iodinations"[All Fields] OR "iodine"[Supplementary Concept] OR "iodine"[All Fields] OR "iodine"[MeSH Terms] OR "iodines"[All Fields]) OR ("silicon"[Supplementary Concept] OR "silicon"[All Fields] OR "silicon"[MeSH Terms] OR "silicon s"[All Fields] OR "silicons"[All Fields])) AND ("neurodevelopment"[All Fields] OR "brain development"[All Fields] OR "motor development"[All Fields] OR "psychomotor development"[All Fields] OR "language development"[All Fields] OR "cognitive development"[All Fields] OR ("cognition"[MeSH Terms] OR "cognition"[All Fields] OR "cognitions"[All Fields] OR "cognitive"[All Fields] OR "cognitively"[All Fields] OR "cognitives"[All Fields]))) NOT ("toxic"[All Fields] OR "toxical"[All Fields] OR "toxically"[All Fields] OR "toxicant"[All Fields] OR "toxicant s"[All Fields] OR "toxicants"[All Fields] OR "toxicated"[All Fields] OR "toxication"[All Fields] OR "toxicities"[All Fields] OR "toxicity"[MeSH Subheading] OR "toxicity"[All Fields] OR "toxicity s"[All Fields] OR "toxics"[All Fields])) NOT ("genes"[MeSH Terms] OR "genes"[All Fields] OR "gene"[All Fields])) NOT ("autism s"[All Fields] OR "autisms"[All Fields] OR "autistic disorder"[MeSH Terms] OR ("autistic"[All Fields] AND "disorder"[All Fields]) OR "autistic disorder"[All Fields] OR "autism"[All Fields])) NOT ("preterm"[All Fields] OR "preterms"[All Fields])) AND ("South Asia"[All Fields] OR ("india"[MeSH Terms] OR "india"[All Fields] OR "india s"[All Fields] OR "indias"[All Fields]) OR ("bangladesh"[MeSH Terms] OR "bangladesh"[All Fields] OR "bangladesh s"[All Fields]) OR ("nepal"[MeSH Terms] OR "nepal"[All Fields] OR "nepal s"[All Fields]) OR ("bhutan"[MeSH Terms] OR "bhutan"[All Fields] OR "bhutan s"[All Fields]) OR "Sri Lanka"[All Fields] OR ("pakistan"[MeSH Terms] OR "pakistan"[All Fields] OR "pakistan s"[All Fields]) OR ("maldive"[All Fields] OR "maldives"[MeSH Terms] OR "maldives"[All Fields]))) AND ((adaptiveclinicaltrial[Filter] OR clinicaltrial[Filter] OR clinicaltrialphasei[Filter] OR clinicaltrialphaseii[Filter] OR clinicaltrialphaseiii[Filter] OR clinicaltrialphaseiv[Filter] OR clinicaltrialprotocol[Filter] OR comparativestudy[Filter] OR controlledclinicaltrial[Filter] OR englishabstract[Filter] OR evaluationstudy[Filter] OR meta-analysis[Filter] OR multicenterstudy[Filter] OR networkmetaanalysis[Filter] OR observationalstudy[Filter] OR pragmaticclinicaltrial[Filter] OR preprint[Filter] OR randomizedcontrolledtrial[Filter] OR researchsupportamericanrecoveryandreinvestmentact[Filter] OR researchsupportnihextramural[Filter] OR researchsupportnihintramural[Filter] OR researchsupportnonusgovt[Filter] OR researchsupportusgovernment[Filter] OR researchsupportusgovtnonphs[Filter] OR researchsupportusgovtphs[Filter] OR retractedpublication[Filter] OR retractionofpublication[Filter] OR review[Filter] OR scientificintegrityreview[Filter] OR scopingreview[Filter] OR systematicreview[Filter] OR technicalreport[Filter] OR twinstudy[Filter] OR validationstudy[Filter]) AND (humans[Filter]) AND (english[Filter]))	
Search 3B	AND (Prevalence OR	((((((("pregnancy"[MeSH Terms] OR "pregnancy"[All Fields] OR "pregnancies"[All Fields] OR "pregnancy s"[All Fields] OR ("maternally"[All Fields] OR "maternities"[All	64

	Epidemiology OR Incidence OR Demography) AND (factors OR determinants OR etiology OR "Socio-Demographic")	Fields] OR "maternity"[All Fields] OR "mothers"[MeSH Terms] OR "mothers"[All Fields] OR "maternal"[All Fields])) AND ("infant"[MeSH Terms] OR "infant"[All Fields] OR "infants"[All Fields] OR "infant s"[All Fields] OR ("offspring"[All Fields] OR "offspring s"[All Fields] OR "offsprings"[All Fields]) OR ("child"[MeSH Terms] OR "child"[All Fields] OR "children"[All Fields] OR "child s"[All Fields] OR "children s"[All Fields] OR "childrens"[All Fields] OR "childs"[All Fields])) AND ((("micronutrients"[Pharmacological Action] OR "micronutrients"[Supplementary Concept] OR "micronutrients"[All Fields] OR "micronutrients"[MeSH Terms] OR "micronutriments"[All Fields] OR "trace elements"[Pharmacological Action] OR "trace elements"[Supplementary Concept] OR "trace elements"[All Fields] OR "micronutrient"[All Fields] OR "trace elements"[MeSH Terms] OR ("trace"[All Fields] AND "elements"[All Fields])) AND "status"[All Fields]) OR (("micronutrients"[Pharmacological Action] OR "micronutrients"[Supplementary Concept] OR "micronutrients"[All Fields] OR "micronutrients"[MeSH Terms] OR "micronutriments"[All Fields] OR "trace elements"[Pharmacological Action] OR "trace elements"[Supplementary Concept] OR "trace elements"[All Fields] OR "micronutrient"[All Fields] OR "trace elements"[MeSH Terms] OR ("trace"[All Fields] AND "elements"[All Fields])) AND ("deficiences"[All Fields] OR "deficiencies"[All Fields] OR "deficiency"[MeSH Subheading] OR "deficiency"[All Fields] OR "deficient"[All Fields] OR "deficients"[All Fields])) OR ("vitamin a"[Supplementary Concept] OR "vitamin a"[All Fields] OR "vitamin a"[MeSH Terms]) OR ("vitamin b complex"[Pharmacological Action] OR "vitamin b complex"[Supplementary Concept] OR "vitamin b complex"[All Fields] OR "vitamin b"[All Fields] OR "vitamin b complex"[MeSH Terms]) OR ("ascorbic acid"[Supplementary Concept] OR "ascorbic acid"[All Fields] OR "vitamin c"[All Fields] OR "ascorbic acid"[MeSH Terms] OR ("ascorbic"[All Fields] AND "acid"[All Fields])) OR ("vitamin d"[Supplementary Concept] OR "vitamin d"[All Fields] OR "ergocalciferols"[Supplementary Concept] OR "ergocalciferols"[All Fields] OR "vitamin d"[MeSH Terms] OR "ergocalciferols"[MeSH Terms]) OR ("vitamin e"[Supplementary Concept] OR "vitamin e"[All Fields] OR "vitamin e"[MeSH Terms]) OR ("vitamin k"[Supplementary Concept] OR "vitamin k"[All Fields] OR "vitamin k"[MeSH Terms]) OR ("thiamine"[Supplementary Concept] OR "thiamine"[All Fields] OR "thiamin"[All Fields] OR "thiamine"[MeSH Terms] OR "thiamines"[All Fields]) OR ("riboflavin"[Supplementary Concept] OR "riboflavin"[All Fields] OR "riboflavin"[MeSH Terms] OR "riboflavine"[All Fields] OR "riboflavins"[All Fields]) OR ("niacin"[Supplementary Concept] OR "niacin"[All Fields] OR "niacin"[MeSH Terms] OR "niacin s"[All Fields] OR "niacinate"[All Fields] OR "niacine"[All Fields] OR "niacins"[All Fields]) OR "B5"[All Fields] OR "B6"[All Fields] OR ("biotin"[Supplementary Concept] OR "biotin"[All Fields] OR "biotin"[MeSH Terms] OR "biotine"[All Fields] OR "biotinated"[All Fields] OR "biotins"[All Fields]) OR ("folic acid"[Supplementary Concept] OR "folic acid"[All Fields] OR "folic acid"[MeSH Terms]	

		OR ("folic"[All Fields] AND "acid"[All Fields])) OR "cobalmin"[All Fields] OR ("iron"[Supplementary Concept] OR "iron"[All Fields] OR "iron"[MeSH Terms]) OR ("calcium"[Supplementary Concept] OR "calcium"[All Fields] OR "calcium"[MeSH Terms] OR "calciums"[All Fields] OR "calcium s"[All Fields]) OR ("sulfur"[Supplementary Concept] OR "sulfur"[All Fields] OR "sulfurs"[All Fields] OR "sulfur"[MeSH Terms] OR "sulfur s"[All Fields] OR "sulfurated"[All Fields] OR "sulfuration"[All Fields] OR "sulfuric"[All Fields] OR "sulfurization"[All Fields] OR "sulfurize"[All Fields] OR "sulfurized"[All Fields] OR "sulfurizing"[All Fields] OR "sulfurous"[All Fields] OR "sulphur"[All Fields] OR "sulphurated"[All Fields] OR "sulphuric"[All Fields] OR "sulphurous"[All Fields] OR "sulphurs"[All Fields]) OR ("magnesium"[Supplementary Concept] OR "magnesium"[All Fields] OR "magnesium"[MeSH Terms] OR "magnesium s"[All Fields] OR "magnesiums"[All Fields]) OR ("phosphorus"[Supplementary Concept] OR "phosphorus"[All Fields] OR "phosphorus"[MeSH Terms]) OR ("sodium"[Supplementary Concept] OR "sodium"[All Fields] OR "sodium"[MeSH Terms] OR "sodiums"[All Fields]) OR ("potassium dietary"[Supplementary Concept] OR "potassium dietary"[All Fields] OR "potassium"[All Fields] OR "potassium"[Supplementary Concept] OR "potassium, dietary"[MeSH Terms] OR ("potassium"[All Fields] AND "dietary"[All Fields]) OR "dietary potassium"[All Fields] OR "potassium"[MeSH Terms]) OR ("zinc"[Supplementary Concept] OR "zinc"[All Fields] OR "zinc"[MeSH Terms]) OR ("copper"[Supplementary Concept] OR "copper"[All Fields] OR "copper"[MeSH Terms] OR "coppers"[All Fields] OR "copper s"[All Fields]) OR ("manganese"[Supplementary Concept] OR "manganese"[All Fields] OR "manganese"[MeSH Terms] OR "manganeses"[All Fields]) OR ("molybdenum"[Supplementary Concept] OR "molybdenum"[All Fields] OR "molybdenum"[MeSH Terms]) OR ("boron"[Supplementary Concept] OR "boron"[All Fields] OR "boron"[MeSH Terms] OR "boron s"[All Fields] OR "boronate"[All Fields] OR "boronated"[All Fields] OR "boronates"[All Fields] OR "boronation"[All Fields] OR "boronic"[All Fields] OR "borons"[All Fields]) OR ("chlorin"[Supplementary Concept] OR "chlorin"[All Fields] OR "chlorinate"[All Fields] OR "chlorinated"[All Fields] OR "chlorinates"[All Fields] OR "chlorinating"[All Fields] OR "chlorinations"[All Fields] OR "chlorinator"[All Fields] OR "chlorinators"[All Fields] OR "chlorine"[Supplementary Concept] OR "chlorine"[All Fields] OR "chlorine"[MeSH Terms] OR "chlorine s"[All Fields] OR "chlorines"[All Fields] OR "chlorins"[All Fields] OR "halogenation"[MeSH Terms] OR "halogenation"[All Fields] OR "chlorination"[All Fields]) OR ("chlorid"[All Fields] OR "chlorides"[Supplementary Concept] OR "chlorides"[All Fields] OR "chloride"[All Fields] OR "chlorides"[MeSH Terms]) OR ("selenium"[Supplementary Concept] OR "selenium"[All Fields] OR "selenium"[MeSH Terms] OR "selenium s"[All Fields] OR "seleniums"[All Fields]) OR ("cobalt"[Supplementary Concept] OR "cobalt"[All Fields] OR "cobalt"[MeSH Terms] OR "cobaltate"[All Fields] OR "cobaltates"[All Fields] OR "cobaltic"[All Fields] OR "cobaltous"[All Fields]) OR	

		("fluorinate"[All Fields] OR "fluorinated"[All Fields] OR "fluorinates"[All Fields] OR "fluorinating"[All Fields] OR "fluorinations"[All Fields] OR "fluorinative"[All Fields] OR "fluorine"[Supplementary Concept] OR "fluorine"[All Fields] OR "fluorine"[MeSH Terms] OR "fluorines"[All Fields] OR "fluorine s"[All Fields] OR "halogenation"[MeSH Terms] OR "halogenation"[All Fields] OR "fluorination"[All Fields]) OR ("fluoridate"[All Fields] OR "fluoridated"[All Fields] OR "fluoridating"[All Fields] OR "fluoridation"[MeSH Terms] OR "fluoridation"[All Fields] OR "fluoridation s"[All Fields] OR "fluoride s"[All Fields] OR "fluorided"[All Fields] OR "fluorides"[Supplementary Concept] OR "fluorides"[All Fields] OR "fluoride"[All Fields] OR "fluorides"[MeSH Terms] OR "fluoridization"[All Fields] OR "fluoridized"[All Fields]) OR ("halogenation"[MeSH Terms] OR "halogenation"[All Fields] OR "iodination"[All Fields] OR "iodin"[All Fields] OR "iodinate"[All Fields] OR "iodinated"[All Fields] OR "iodinates"[All Fields] OR "iodinating"[All Fields] OR "iodinations"[All Fields] OR "iodine"[Supplementary Concept] OR "iodine"[All Fields] OR "iodine"[MeSH Terms] OR "iodines"[All Fields]) OR ("silicon"[Supplementary Concept] OR "silicon"[All Fields] OR "silicon"[MeSH Terms] OR "silicon s"[All Fields] OR "silicons"[All Fields])) AND ("neurodevelopment"[All Fields] OR "brain development"[All Fields] OR "motor development"[All Fields] OR "psychomotor development"[All Fields] OR "language development"[All Fields] OR "cognitive development"[All Fields] OR ("cognition"[MeSH Terms] OR "cognition"[All Fields] OR "cognitions"[All Fields] OR "cognitive"[All Fields] OR "cognitively"[All Fields] OR "cognitives"[All Fields]))) NOT ("toxic"[All Fields] OR "toxical"[All Fields] OR "toxically"[All Fields] OR "toxicant"[All Fields] OR "toxicant s"[All Fields] OR "toxicants"[All Fields] OR "toxicated"[All Fields] OR "toxication"[All Fields] OR "toxicities"[All Fields] OR "toxicity"[MeSH Subheading] OR "toxicity"[All Fields] OR "toxicity s"[All Fields] OR "toxics"[All Fields])) NOT ("genes"[MeSH Terms] OR "genes"[All Fields] OR "gene"[All Fields])) NOT ("autism s"[All Fields] OR "autisms"[All Fields] OR "autistic disorder"[MeSH Terms] OR ("autistic"[All Fields] AND "disorder"[All Fields]) OR "autistic disorder"[All Fields] OR "autism"[All Fields])) NOT ("preterm"[All Fields] OR "preterms"[All Fields])) AND ("South Asia"[All Fields] OR ("india"[MeSH Terms] OR "india"[All Fields] OR "india s"[All Fields] OR "indias"[All Fields]) OR ("bangladesh"[MeSH Terms] OR "bangladesh"[All Fields] OR "bangladesh s"[All Fields]) OR ("nepal"[MeSH Terms] OR "nepal"[All Fields] OR "nepal s"[All Fields]) OR ("bhutan"[MeSH Terms] OR "bhutan"[All Fields] OR "bhutan s"[All Fields]) OR "Sri Lanka"[All Fields] OR ("pakistan"[MeSH Terms] OR "pakistan"[All Fields] OR "pakistan s"[All Fields]) OR ("maldive"[All Fields] OR "maldives"[MeSH Terms] OR "maldives"[All Fields])) AND ("epidemiology"[MeSH Subheading] OR "epidemiology"[All Fields] OR "prevalence"[All Fields] OR "prevalence"[MeSH Terms] OR "prevalance"[All Fields] OR "prevalences"[All Fields] OR "prevalence s"[All Fields] OR "prevalent"[All Fields] OR "prevalently"[All Fields] OR "prevalents"[All Fields] OR ("epidemiologies"[All Fields] OR "epidemiology"[MeSH Subheading] OR	

		"epidemiology"[All Fields] OR "epidemiology"[MeSH Terms] OR "epidemiology s"[All Fields]) OR ("epidemiology"[MeSH Subheading] OR "epidemiology"[All Fields] OR "incidence"[All Fields] OR "incidence"[MeSH Terms] OR "incidences"[All Fields] OR "incident"[All Fields] OR "incidents"[All Fields]) OR ("demographies"[All Fields] OR "demography"[MeSH Terms] OR "demography"[All Fields])) AND ("factor"[All Fields] OR "factor s"[All Fields] OR "factors"[All Fields] OR ("analysis"[MeSH Subheading] OR "analysis"[All Fields] OR "determination"[All Fields] OR "determinant"[All Fields] OR "determinants"[All Fields] OR "determinate"[All Fields] OR "determinated"[All Fields] OR "determinates"[All Fields] OR "determinating"[All Fields] OR "determinations"[All Fields] OR "determine"[All Fields] OR "determined"[All Fields] OR "determines"[All Fields] OR "determining"[All Fields]) OR ("aetiologie"[All Fields] OR "aetiologies"[All Fields] OR "aetiology"[All Fields] OR "etiologies"[All Fields] OR "etiology"[MeSH Subheading] OR "etiology"[All Fields] OR "causality"[MeSH Terms] OR "causality"[All Fields]) OR "Socio-Demographic"[All Fields])) AND ((humans[Filter]) AND (english[Filter]))<br>Translations<br>pregnancy: "pregnancy"[MeSH Terms] OR "pregnancy"[All Fields] OR "pregnancies"[All Fields] OR "pregnancy's"[All Fields]<br>maternal: "maternally"[All Fields] OR "maternities"[All Fields] OR "maternity"[All Fields] OR "mothers"[MeSH Terms] OR "mothers"[All Fields] OR "maternal"[All Fields]<br>infant: "infant"[MeSH Terms] OR "infant"[All Fields] OR "infants"[All Fields] OR "infant's"[All Fields]<br>offspring: "offspring"[All Fields] OR "offspring's"[All Fields] OR "offsprings"[All Fields]<br>child: "child"[MeSH Terms] OR "child"[All Fields] OR "children"[All Fields] OR "child's"[All Fields] OR "children's"[All Fields] OR "childrens"[All Fields] OR "childs"[All Fields]<br>micronutrient: "micronutrients"[Pharmacological Action] OR "micronutrients"[Supplementary Concept] OR "micronutrients"[All Fields] OR "micronutrients"[MeSH Terms] OR "micronutriments"[All Fields] OR "trace elements"[Pharmacological Action] OR "trace elements"[Supplementary Concept] OR "trace elements"[All Fields] OR "micronutrient"[All Fields] OR "trace elements"[MeSH Terms] OR ("trace"[All Fields] AND "elements"[All Fields])<br>micronutrient: "micronutrients"[Pharmacological Action] OR "micronutrients"[Supplementary Concept] OR "micronutrients"[All Fields] OR "micronutrients"[MeSH Terms] OR "micronutriments"[All Fields] OR "trace elements"[Pharmacological Action] OR "trace elements"[Supplementary Concept] OR "trace elements"[All Fields] OR "micronutrient"[All Fields] OR "trace elements"[MeSH Terms] OR ("trace"[All Fields] AND "elements"[All Fields])	

		deficiency: "deficiences"[All Fields] OR "deficiencies"[All Fields] OR "deficiency"[Subheading] OR "deficiency"[All Fields] OR "deficient"[All Fields] OR "deficients"[All Fields]<br>vitamin A: "vitamin a"[Supplementary Concept] OR "vitamin a"[All Fields] OR "vitamin a"[MeSH Terms]<br>vitamin B: "vitamin b complex"[Pharmacological Action] OR "vitamin b complex"[Supplementary Concept] OR "vitamin b complex"[All Fields] OR "vitamin b"[All Fields] OR "vitamin b complex"[MeSH Terms]<br>vitamin C: "ascorbic acid"[Supplementary Concept] OR "ascorbic acid"[All Fields] OR "vitamin c"[All Fields] OR "ascorbic acid"[MeSH Terms] OR ("ascorbic"[All Fields] AND "acid"[All Fields])<br>vitamin D: "vitamin d"[Supplementary Concept] OR "vitamin d"[All Fields] OR "ergocalciferols"[Supplementary Concept] OR "ergocalciferols"[All Fields] OR "vitamin d"[MeSH Terms] OR "ergocalciferols"[MeSH Terms]<br>vitamin E: "vitamin e"[Supplementary Concept] OR "vitamin e"[All Fields] OR "vitamin e"[MeSH Terms]<br>vitamin K: "vitamin k"[Supplementary Concept] OR "vitamin k"[All Fields] OR "vitamin k"[MeSH Terms]<br>thiamin: "thiamine"[Supplementary Concept] OR "thiamine"[All Fields] OR "thiamin"[All Fields] OR "thiamine"[MeSH Terms] OR "thiamines"[All Fields]<br>riboflavin: "riboflavin"[Supplementary Concept] OR "riboflavin"[All Fields] OR "riboflavin"[MeSH Terms] OR "riboflavine"[All Fields] OR "riboflavins"[All Fields]<br>niacin: "niacin"[Supplementary Concept] OR "niacin"[All Fields] OR "niacin"[MeSH Terms] OR "niacin's"[All Fields] OR "niacinate"[All Fields] OR "niacine"[All Fields] OR "niacins"[All Fields]<br>biotin: "biotin"[Supplementary Concept] OR "biotin"[All Fields] OR "biotin"[MeSH Terms] OR "biotine"[All Fields] OR "biotinated"[All Fields] OR "biotins"[All Fields]<br>folic acid: "folic acid"[Supplementary Concept] OR "folic acid"[All Fields] OR "folic acid"[MeSH Terms] OR ("folic"[All Fields] AND "acid"[All Fields])<br>Iron: "iron"[Supplementary Concept] OR "iron"[All Fields] OR "iron"[MeSH Terms]<br>calcium: "calcium"[Supplementary Concept] OR "calcium"[All Fields] OR "calcium"[MeSH Terms] OR "calciums"[All Fields] OR "calcium's"[All Fields]<br>sulfur: "sulfur"[Supplementary Concept] OR "sulfur"[All Fields] OR "sulfurs"[All Fields] OR "sulfur"[MeSH Terms] OR "sulfur's"[All Fields] OR "sulfurated"[All Fields] OR "sulfuration"[All Fields] OR "sulfuric"[All Fields] OR "sulfurization"[All Fields] OR "sulfurize"[All Fields] OR "sulfurized"[All Fields] OR "sulfurizing"[All Fields] OR "sulfurous"[All Fields] OR "sulphur"[All Fields] OR "sulphurated"[All Fields] OR "sulphuric"[All Fields] OR "sulphurous"[All Fields] OR "sulphurs"[All Fields]<br>magnesium: "magnesium"[Supplementary Concept] OR "magnesium"[All Fields] OR "magnesium"[MeSH Terms] OR "magnesium's"[All Fields] OR "magnesiums"[All Fields]	

		phosphorus: "phosphorus"[Supplementary Concept] OR "phosphorus"[All Fields] OR "phosphorus"[MeSH Terms]<br>sodium: "sodium"[Supplementary Concept] OR "sodium"[All Fields] OR "sodium"[MeSH Terms] OR "sodiums"[All Fields]<br>potassium: "potassium, dietary"[Supplementary Concept] OR "potassium, dietary"[All Fields] OR "potassium"[All Fields] OR "potassium"[Supplementary Concept] OR "potassium, dietary"[MeSH Terms] OR ("potassium"[All Fields] AND "dietary"[All Fields]) OR "dietary potassium"[All Fields] OR "potassium"[MeSH Terms]<br>zinc: "zinc"[Supplementary Concept] OR "zinc"[All Fields] OR "zinc"[MeSH Terms]<br>copper: "copper"[Supplementary Concept] OR "copper"[All Fields] OR "copper"[MeSH Terms] OR "coppers"[All Fields] OR "copper's"[All Fields]<br>manganese: "manganese"[Supplementary Concept] OR "manganese"[All Fields] OR "manganese"[MeSH Terms] OR "manganeses"[All Fields]<br>molybdenum: "molybdenum"[Supplementary Concept] OR "molybdenum"[All Fields] OR "molybdenum"[MeSH Terms]<br>boron: "boron"[Supplementary Concept] OR "boron"[All Fields] OR "boron"[MeSH Terms] OR "boron's"[All Fields] OR "boronate"[All Fields] OR "boronated"[All Fields] OR "boronates"[All Fields] OR "boronation"[All Fields] OR "boronic"[All Fields] OR "borons"[All Fields]<br>chlorine: "chlorin"[Supplementary Concept] OR "chlorin"[All Fields] OR "chlorinate"[All Fields] OR "chlorinated"[All Fields] OR "chlorinates"[All Fields] OR "chlorinating"[All Fields] OR "chlorinations"[All Fields] OR "chlorinator"[All Fields] OR "chlorinators"[All Fields] OR "chlorine"[Supplementary Concept] OR "chlorine"[All Fields] OR "chlorine"[MeSH Terms] OR "chlorine's"[All Fields] OR "chlorines"[All Fields] OR "chlorins"[All Fields] OR "halogenation"[MeSH Terms] OR "halogenation"[All Fields] OR "chlorination"[All Fields]<br>chloride: "chlorid"[All Fields] OR "chlorides"[Supplementary Concept] OR "chlorides"[All Fields] OR "chloride"[All Fields] OR "chlorides"[MeSH Terms]<br>selenium: "selenium"[Supplementary Concept] OR "selenium"[All Fields] OR "selenium"[MeSH Terms] OR "selenium's"[All Fields] OR "seleniums"[All Fields]<br>cobalt: "cobalt"[Supplementary Concept] OR "cobalt"[All Fields] OR "cobalt"[MeSH Terms] OR "cobaltate"[All Fields] OR "cobaltates"[All Fields] OR "cobaltic"[All Fields] OR "cobaltous"[All Fields]<br>fluorine: "fluorinate"[All Fields] OR "fluorinated"[All Fields] OR "fluorinates"[All Fields] OR "fluorinating"[All Fields] OR "fluorinations"[All Fields] OR "fluorinative"[All Fields] OR "fluorine"[Supplementary Concept] OR "fluorine"[All Fields] OR "fluorine"[MeSH Terms] OR "fluorines"[All Fields] OR "fluorine's"[All Fields] OR "halogenation"[MeSH Terms] OR "halogenation"[All Fields] OR "fluorination"[All Fields]<br>fluoride: "fluoridate"[All Fields] OR "fluoridated"[All Fields] OR "fluoridating"[All Fields] OR "fluoridation"[MeSH Terms] OR "fluoridation"[All Fields] OR	

		"fluoridation's"[All Fields] OR "fluoride's"[All Fields] OR "fluorided"[All Fields] OR "fluorides"[Supplementary Concept] OR "fluorides"[All Fields] OR "fluoride"[All Fields] OR "fluorides"[MeSH Terms] OR "fluoridization"[All Fields] OR "fluoridized"[All Fields]<br>iodine: "halogenation"[MeSH Terms] OR "halogenation"[All Fields] OR "iodination"[All Fields] OR "iodin"[All Fields] OR "iodinate"[All Fields] OR "iodinated"[All Fields] OR "iodinates"[All Fields] OR "iodinating"[All Fields] OR "iodinations"[All Fields] OR "iodine"[Supplementary Concept] OR "iodine"[All Fields] OR "iodine"[MeSH Terms] OR "iodines"[All Fields]<br>silicon: "silicon"[Supplementary Concept] OR "silicon"[All Fields] OR "silicon"[MeSH Terms] OR "silicon's"[All Fields] OR "silicons"[All Fields]<br>Cognition: "cognition"[MeSH Terms] OR "cognition"[All Fields] OR "cognitions"[All Fields] OR "cognitive"[All Fields] OR "cognitively"[All Fields] OR "cognitives"[All Fields]<br>toxic: "toxic"[All Fields] OR "toxical"[All Fields] OR "toxically"[All Fields] OR "toxicant"[All Fields] OR "toxicant's"[All Fields] OR "toxicants"[All Fields] OR "toxicated"[All Fields] OR "toxication"[All Fields] OR "toxicities"[All Fields] OR "toxicity"[Subheading] OR "toxicity"[All Fields] OR "toxicity's"[All Fields] OR "toxics"[All Fields]<br>gene: "genes"[MeSH Terms] OR "genes"[All Fields] OR "gene"[All Fields]<br>autism: "autism's"[All Fields] OR "autisms"[All Fields] OR "autistic disorder"[MeSH Terms] OR ("autistic"[All Fields] AND "disorder"[All Fields]) OR "autistic disorder"[All Fields] OR "autism"[All Fields]<br>preterm: "preterm"[All Fields] OR "preterms"[All Fields]<br>India: "india"[MeSH Terms] OR "india"[All Fields] OR "india's"[All Fields] OR "indias"[All Fields]<br>Bangladesh: "bangladesh"[MeSH Terms] OR "bangladesh"[All Fields] OR "bangladesh's"[All Fields]<br>Nepal: "nepal"[MeSH Terms] OR "nepal"[All Fields] OR "nepal's"[All Fields]<br>Bhutan: "bhutan"[MeSH Terms] OR "bhutan"[All Fields] OR "bhutan's"[All Fields]<br>Pakistan: "pakistan"[MeSH Terms] OR "pakistan"[All Fields] OR "pakistan's"[All Fields]<br>Maldives: "maldive"[All Fields] OR "maldives"[MeSH Terms] OR "maldives"[All Fields]<br>Prevalence: "epidemiology"[Subheading] OR "epidemiology"[All Fields] OR "prevalence"[All Fields] OR "prevalence"[MeSH Terms] OR "prevalance"[All Fields] OR "prevalences"[All Fields] OR "prevalence's"[All Fields] OR "prevalent"[All Fields] OR "prevalently"[All Fields] OR "prevalents"[All Fields]<br>Epidemiology: "epidemiologies"[All Fields] OR "epidemiology"[Subheading] OR "epidemiology"[All Fields] OR "epidemiology"[MeSH Terms] OR "epidemiology's"[All Fields]	

		Incidence: "epidemiology"[Subheading] OR "epidemiology"[All Fields] OR "incidence"[All Fields] OR "incidence"[MeSH Terms] OR "incidences"[All Fields] OR "incident"[All Fields] OR "incidents"[All Fields]<br>Demography: "demographies"[All Fields] OR "demography"[MeSH Terms] OR "demography"[All Fields]<br>factors: "factor"[All Fields] OR "factor's"[All Fields] OR "factors"[All Fields]<br>determinants: "analysis"[Subheading] OR "analysis"[All Fields] OR "determination"[All Fields] OR "determinant"[All Fields] OR "determinants"[All Fields] OR "determinate"[All Fields] OR "determinated"[All Fields] OR "determinates"[All Fields] OR "determinating"[All Fields] OR "determinations"[All Fields] OR "determine"[All Fields] OR "determined"[All Fields] OR "determines"[All Fields] OR "determining"[All Fields]<br>etiology: "aetiologie"[All Fields] OR "aetiologies"[All Fields] OR "aetiology"[All Fields] OR "etiologies"[All Fields] OR "etiology"[Subheading] OR "etiology"[All Fields] OR "causality"[MeSH Terms] OR "causality"[All Fields]	
Search 4	Adaptive Clinical Trial, Clinical Trial, Clinical Trial Protocol, Clinical Trial, Phase I, Clinical Trial, Phase II, Clinical Trial, Phase III, Clinical Trial, Phase IV, Comparative Study, Controlled Clinical Trial, English Abstract, Multicenter Study, Pragmatic Clinical Trial, Preprint, Randomized Controlled Trial,	((((((("pregnancy"[MeSH Terms] OR "pregnancy"[All Fields] OR "pregnancies"[All Fields] OR "pregnancy s"[All Fields] OR ("maternally"[All Fields] OR "maternities"[All Fields] OR "maternity"[All Fields] OR "mothers"[MeSH Terms] OR "mothers"[All Fields] OR "maternal"[All Fields])) AND ("infant"[MeSH Terms] OR "infant"[All Fields] OR "infants"[All Fields] OR "infant s"[All Fields] OR ("offspring"[All Fields] OR "offspring s"[All Fields] OR "offsprings"[All Fields]) OR ("child"[MeSH Terms] OR "child"[All Fields] OR "children"[All Fields] OR "child s"[All Fields] OR "children s"[All Fields] OR "childrens"[All Fields] OR "childs"[All Fields])) AND ((("micronutrients"[Pharmacological Action] OR "micronutrients"[Supplementary Concept] OR "micronutrients"[All Fields] OR "micronutrients"[MeSH Terms] OR "micronutriments"[All Fields] OR "trace elements"[Pharmacological Action] OR "trace elements"[Supplementary Concept] OR "trace elements"[All Fields] OR "micronutrient"[All Fields] OR "trace elements"[MeSH Terms] OR ("trace"[All Fields] AND "elements"[All Fields])) AND "status"[All Fields]) OR (("micronutrients"[Pharmacological Action] OR "micronutrients"[Supplementary Concept] OR "micronutrients"[All Fields] OR "micronutrients"[MeSH Terms] OR "micronutriments"[All Fields] OR "trace elements"[Pharmacological Action] OR "trace elements"[Supplementary Concept] OR "trace elements"[All Fields] OR "micronutrient"[All Fields] OR "trace elements"[MeSH Terms] OR ("trace"[All Fields] AND "elements"[All Fields])) AND ("deficiences"[All Fields] OR "deficiencies"[All Fields] OR "deficiency"[MeSH Subheading] OR "deficiency"[All Fields] OR "deficient"[All Fields] OR "deficients"[All Fields])) OR ("vitamin a"[Supplementary Concept] OR "vitamin a"[All Fields] OR "vitamin a"[MeSH Terms]) OR ("vitamin b complex"[Pharmacological Action] OR "vitamin b complex"[Supplementary Concept] OR "vitamin b complex"[All Fields] OR "vitamin b"[All Fields] OR "vitamin b complex"[MeSH Terms]) OR ("ascorbic acid"[Supplementary Concept] OR "ascorbic acid"[All Fields] OR	81

	Research Support, N.I.H., Extramural, Research Support, N.I.H., Intramural, Research Support, Non-U.S. Gov't, Research Support, U.S. Gov't, Non-P.H.S., Research Support, U.S. Gov't, P.H.S., Research Support, U.S. Gov't, Retracted Publication, Retraction of Publication, English, Humans	"vitamin c"[All Fields] OR "ascorbic acid"[MeSH Terms] OR ("ascorbic"[All Fields] AND "acid"[All Fields])) OR ("vitamin d"[Supplementary Concept] OR "vitamin d"[All Fields] OR "ergocalciferols"[Supplementary Concept] OR "ergocalciferols"[All Fields] OR "vitamin d"[MeSH Terms] OR "ergocalciferols"[MeSH Terms]) OR ("vitamin e"[Supplementary Concept] OR "vitamin e"[All Fields] OR "vitamin e"[MeSH Terms]) OR ("vitamin k"[Supplementary Concept] OR "vitamin k"[All Fields] OR "vitamin k"[MeSH Terms]) OR ("thiamine"[Supplementary Concept] OR "thiamine"[All Fields] OR "thiamin"[All Fields] OR "thiamine"[MeSH Terms] OR "thiamines"[All Fields]) OR ("riboflavin"[Supplementary Concept] OR "riboflavin"[All Fields] OR "riboflavin"[MeSH Terms] OR "riboflavine"[All Fields] OR "riboflavins"[All Fields]) OR ("niacin"[Supplementary Concept] OR "niacin"[All Fields] OR "niacin"[MeSH Terms] OR "niacin s"[All Fields] OR "niacinate"[All Fields] OR "niacine"[All Fields] OR "niacins"[All Fields]) OR "B5"[All Fields] OR "B6"[All Fields] OR ("biotin"[Supplementary Concept] OR "biotin"[All Fields] OR "biotin"[MeSH Terms] OR "biotine"[All Fields] OR "biotinated"[All Fields] OR "biotins"[All Fields]) OR ("folic acid"[Supplementary Concept] OR "folic acid"[All Fields] OR "folic acid"[MeSH Terms] OR ("folic"[All Fields] AND "acid"[All Fields])) OR "cobalmin"[All Fields] OR ("iron"[Supplementary Concept] OR "iron"[All Fields] OR "iron"[MeSH Terms]) OR ("calcium"[Supplementary Concept] OR "calcium"[All Fields] OR "calcium"[MeSH Terms] OR "calciums"[All Fields] OR "calcium s"[All Fields]) OR ("sulfur"[Supplementary Concept] OR "sulfur"[All Fields] OR "sulfurs"[All Fields] OR "sulfur"[MeSH Terms] OR "sulfur s"[All Fields] OR "sulfurated"[All Fields] OR "sulfuration"[All Fields] OR "sulfuric"[All Fields] OR "sulfurization"[All Fields] OR "sulfurize"[All Fields] OR "sulfurized"[All Fields] OR "sulfurizing"[All Fields] OR "sulfurous"[All Fields] OR "sulphur"[All Fields] OR "sulphurated"[All Fields] OR "sulphuric"[All Fields] OR "sulphurous"[All Fields] OR "sulphurs"[All Fields]) OR ("magnesium"[Supplementary Concept] OR "magnesium"[All Fields] OR "magnesium"[MeSH Terms] OR "magnesium s"[All Fields] OR "magnesiums"[All Fields]) OR ("phosphorus"[Supplementary Concept] OR "phosphorus"[All Fields] OR "phosphorus"[MeSH Terms]) OR ("sodium"[Supplementary Concept] OR "sodium"[All Fields] OR "sodium"[MeSH Terms] OR "sodiums"[All Fields]) OR ("potassium dietary"[Supplementary Concept] OR "potassium dietary"[All Fields] OR "potassium"[All Fields] OR "potassium"[Supplementary Concept] OR "potassium, dietary"[MeSH Terms] OR ("potassium"[All Fields] AND "dietary"[All Fields]) OR "dietary potassium"[All Fields] OR "potassium"[MeSH Terms]) OR ("zinc"[Supplementary Concept] OR "zinc"[All Fields] OR "zinc"[MeSH Terms]) OR ("copper"[Supplementary Concept] OR "copper"[All Fields] OR "copper"[MeSH Terms] OR "coppers"[All Fields] OR "copper s"[All Fields]) OR ("manganese"[Supplementary Concept] OR "manganese"[All Fields] OR "manganese"[MeSH Terms] OR "manganeses"[All Fields]) OR ("molybdenum"[Supplementary Concept] OR "molybdenum"[All Fields] OR "molybdenum"[MeSH Terms]) OR ("boron"[Supplementary Concept] OR "boron"[All	

		Fields] OR "boron"[MeSH Terms] OR "boron s"[All Fields] OR "boronate"[All Fields] OR "boronated"[All Fields] OR "boronates"[All Fields] OR "boronation"[All Fields] OR "boronic"[All Fields] OR "borons"[All Fields]) OR ("chlorin"[Supplementary Concept] OR "chlorin"[All Fields] OR "chlorinate"[All Fields] OR "chlorinated"[All Fields] OR "chlorinates"[All Fields] OR "chlorinating"[All Fields] OR "chlorinations"[All Fields] OR "chlorinator"[All Fields] OR "chlorinators"[All Fields] OR "chlorine"[Supplementary Concept] OR "chlorine"[All Fields] OR "chlorine"[MeSH Terms] OR "chlorine s"[All Fields] OR "chlorines"[All Fields] OR "chlorins"[All Fields] OR "halogenation"[MeSH Terms] OR "halogenation"[All Fields] OR "chlorination"[All Fields]) OR ("chlorid"[All Fields] OR "chlorides"[Supplementary Concept] OR "chlorides"[All Fields] OR "chloride"[All Fields] OR "chlorides"[MeSH Terms]) OR ("selenium"[Supplementary Concept] OR "selenium"[All Fields] OR "selenium"[MeSH Terms] OR "selenium s"[All Fields] OR "seleniums"[All Fields]) OR ("cobalt"[Supplementary Concept] OR "cobalt"[All Fields] OR "cobalt"[MeSH Terms] OR "cobaltate"[All Fields] OR "cobaltates"[All Fields] OR "cobaltic"[All Fields] OR "cobaltous"[All Fields]) OR ("fluorinate"[All Fields] OR "fluorinated"[All Fields] OR "fluorinates"[All Fields] OR "fluorinating"[All Fields] OR "fluorinations"[All Fields] OR "fluorinative"[All Fields] OR "fluorine"[Supplementary Concept] OR "fluorine"[All Fields] OR "fluorine"[MeSH Terms] OR "fluorines"[All Fields] OR "fluorine s"[All Fields] OR "halogenation"[MeSH Terms] OR "halogenation"[All Fields] OR "fluorination"[All Fields]) OR ("fluoridate"[All Fields] OR "fluoridated"[All Fields] OR "fluoridating"[All Fields] OR "fluoridation"[MeSH Terms] OR "fluoridation"[All Fields] OR "fluoridation s"[All Fields] OR "fluoride s"[All Fields] OR "fluorided"[All Fields] OR "fluorides"[Supplementary Concept] OR "fluorides"[All Fields] OR "fluoride"[All Fields] OR "fluorides"[MeSH Terms] OR "fluoridization"[All Fields] OR "fluoridized"[All Fields]) OR ("halogenation"[MeSH Terms] OR "halogenation"[All Fields] OR "iodination"[All Fields] OR "iodin"[All Fields] OR "iodinate"[All Fields] OR "iodinated"[All Fields] OR "iodinates"[All Fields] OR "iodinating"[All Fields] OR "iodinations"[All Fields] OR "iodine"[Supplementary Concept] OR "iodine"[All Fields] OR "iodine"[MeSH Terms] OR "iodines"[All Fields]) OR ("silicon"[Supplementary Concept] OR "silicon"[All Fields] OR "silicon"[MeSH Terms] OR "silicon s"[All Fields] OR "silicons"[All Fields])) AND ("neurodevelopment"[All Fields] OR "brain development"[All Fields] OR "motor development"[All Fields] OR "psychomotor development"[All Fields] OR "language development"[All Fields] OR "cognitive development"[All Fields] OR ("cognition"[MeSH Terms] OR "cognition"[All Fields] OR "cognitions"[All Fields] OR "cognitive"[All Fields] OR "cognitively"[All Fields] OR "cognitives"[All Fields]))) NOT ("toxic"[All Fields] OR "toxical"[All Fields] OR "toxically"[All Fields] OR "toxicant"[All Fields] OR "toxicant s"[All Fields] OR "toxicants"[All Fields] OR "toxicated"[All Fields] OR "toxication"[All Fields] OR "toxicities"[All Fields] OR "toxicity"[MeSH Subheading] OR "toxicity"[All Fields] OR "toxicity s"[All Fields] OR "toxics"[All Fields])) NOT ("genes"[MeSH Terms] OR "genes"[All Fields] OR "gene"[All Fields])) NOT ("autism	

		s"[All Fields] OR "autisms"[All Fields] OR "autistic disorder"[MeSH Terms] OR ("autistic"[All Fields] AND "disorder"[All Fields]) OR "autistic disorder"[All Fields] OR "autism"[All Fields])) NOT ("preterm"[All Fields] OR "preterms"[All Fields])) AND ("South Asia"[All Fields] OR ("india"[MeSH Terms] OR "india"[All Fields] OR "india s"[All Fields] OR "indias"[All Fields]) OR ("bangladesh"[MeSH Terms] OR "bangladesh"[All Fields] OR "bangladesh s"[All Fields]) OR ("nepal"[MeSH Terms] OR "nepal"[All Fields] OR "nepal s"[All Fields]) OR ("bhutan"[MeSH Terms] OR "bhutan"[All Fields] OR "bhutan s"[All Fields]) OR "Sri Lanka"[All Fields] OR ("pakistan"[MeSH Terms] OR "pakistan"[All Fields] OR "pakistan s"[All Fields]) OR ("maldive"[All Fields] OR "maldives"[MeSH Terms] OR "maldives"[All Fields]))) AND ((adaptiveclinicaltrial[Filter] OR clinicaltrial[Filter] OR clinicaltrialprotocol[Filter] OR clinicaltrialphasei[Filter] OR clinicaltrialphaseii[Filter] OR clinicaltrialphaseiii[Filter] OR clinicaltrialphaseiv[Filter] OR comparativestudy[Filter] OR controlledclinicaltrial[Filter] OR englishabstract[Filter] OR multicenterstudy[Filter] OR pragmaticclinicaltrial[Filter] OR preprint[Filter] OR randomizedcontrolledtrial[Filter] OR researchsupportamericanrecoveryandreinvestmentact[Filter] OR researchsupportnihextramural[Filter] OR researchsupportnihintramural[Filter] OR researchsupportnonusgovt[Filter] OR researchsupportusgovtnonphs[Filter] OR researchsupportusgovtphs[Filter] OR researchsupportusgovernment[Filter] OR retractedpublication[Filter] OR retractionofpublication[Filter]) AND (humans[Filter]) AND (english[Filter]))	

Appendix II: Screening Forms

Study Selection Form for Title/Abstract Screening

A. **General Study Information**

**Study Title**	
**First Author**	
**Year of Publication**	
**Name of the Journal**	
**Abstract Available**	

B) Screening Criteria

1. **Language:** Title/abstract in English
   □ Yes
   □ No
2. **Subjects:** Study includes human participants
   □ Yes
   □ Unclear
   □ No
3. **Study Population:** Involves healthy pregnant women
   □ Yes
   □ Unclear
   □ No
4. **Study Population:** Involves full-term infants (age $\leq$ 2 years)
   □ Yes
   □ Unclear
   □ No
5. **Micronutrient Status:** Mentions maternal micronutrient status/intake/supplementation
   □ Yes
   □ Unclear
   □ No

6. **Neurodevelopmental Outcomes:** – Refers to infant neurodevelopment (e.g., cognitive, motor, language outcomes)
   □ Yes
   □ Unclear
   □ No
7. **Association Established:** – Association of Micronutrient Status with Infant Neurodevelopment
   □ Yes

□ Unclear
□ No

STATUS OF INITIAL SCREENING

□ Include (All 7 criterion questions checked "Yes" or "Unclear")
□ Exclude (Any of the 7 criteria questions checked "No")

Reason for Exclusion: ____

***Reasons for Exclusion**

1. Language other than English
2. Animal studies
3. Wrong population

3A. Either of one population i.e., pregnant women or infants is missing). Exclusion Women with multiple pregnancies and adolescent pregnancies or

3B. Pregnant females with diagnosed chronic illnesses, or infants born extremely pre-term, severe SGA and Neonatal Neural/congenital Disorders)

4. Wrong outcome (No Association of Micronutrient Status with Infant Neurodevelopment)

Study Selection Form for Full-Text Screening

A. General Study Information

**Study Title**	
**First Author**	
**Year of Publication**	
**Name of the Journal**	
**Name of Country**	

B) Screening Criteria

1. Is Full Text available?

□ Yes

□ No

2. Is Full Text available in English?

□Yes

□ No

3. Is the age of the pregnant women between 15-49 years?

□ Yes

□ No

4. Is the age of the infant ≤ 2 years?

□ Yes

□ No

5. **Micronutrient Status:** Does the study assess maternal micronutrient status/ micronutrient dietary intake/micronutrient supplementation during pregnancy? *(biochemical assessment)*

□Yes

□ No

6. **Neurodevelopmental Assessment:** Does the study do neurodevelopmental assessment of infants (≤ 2 years)?

□Yes

□ No

7. **Association Established:** Has the study established a clear relationship linking maternal micronutrient status with infant neurodevelopment?

□Yes

□ No

C) Study Population Exclusion Criteria

8. Is the study conducted **exclusively on adolescent pregnancies** or **multiple gestations**?

□Yes

□ No

9. Does the study enrolled **pregnant women with pre-existing medical conditions** (e.g., chronic diseases, mental health disorders, substance abuse, or undergoing infertility treatment)?

□Yes

□ No

10. Is the study conducted **exclusively on infants born extremely preterm**, **severely small for gestational age (SGA)**, or with **pre-diagnosed neural/congenital disorders**?

□Yes

□ No

STATUS OF FINAL SCREENING

- **Include** (If questions 2- 7 checked "Yes")
- **Exclude** (Any of the question from 2-7 checked "No" OR any of the questions from 8- 10 checked Yes)
- **Pending**
    - (If Q1 answers No)
    - (Closely related study so will be considered for snowballing)

Reason for Exclusion: ____

***Reasons for Exclusion**

1. Language other than English
2. 2A Pregnant Women aged < 15 years or > 49 years.
   2B Women with multiple pregnancies and adolescent pregnancies or pregnant females with diagnosed chronic illnesses, or infants born extremely pre-term, severe SGA and Neonatal Neural/congenital Disorders.
3. Wrong outcome (No Association of Micronutrient Status with Infant Neurodevelopment)

9. Not Applicable

Appendix IV: Data Extraction Form

Data extraction Form Search 1 conducted on global basis										
**S. No**	**Study Identification**	**Study Characteristics**	**Population Characteristics**	**Exposure/ Intervention Details**	**Infant Neurodevelopmental Outcomes**	**Statistical Considerations**	**Key Findings (Factors)**	**Grading and Strength of Evidence**	**Author Reported Limitations**	**Fundings**
	1.Author(s)<br>2.Year of Publication<br>3.Title	1.Study Design<br>2.Type of evidence source<br>3.Country<br>4.Setting of the study	1.Sample Size (Maternal, Infant)<br>2.Maternal Age (Mean/Range)<br>3. Infant Age (Mean/Range)<br>4.Parity<br>5.Socio economic status	1.Name of the Micronutrient(s) supplemented /deficient/studied:<br><br>2.Method of Assessment/ Supplementation<br>3.Timing of Exposure/Intervention<br>a. Dosage and frequency of supplementation<br>b. Duration of supplementation<br>4. Other intervention Specifics	1.Outcome Domains (e.g., cognitive, motor, language)<br>2.Measurement Tools Used<br>3.Timing of Assessment (infant age at evaluation)	1.Statistical Analysis<br><br>2.Confounding variables adjusted				

Data extraction Form for Search 2 focused on South Asian Countries										
**S. No**	**Study Identification**	**Study Characteristics**	**Population Characteristics**	**Exposure/ Intervention Details**	**Infant Neurodevelopmental Outcomes**	**Statistical Considerations**	**Key Findings (Factors)**	**Grading and Strength of Evidence**	**Author Reported Limitations**	**Fundings**
	1.Author(s)<br>2.Year of Publication<br>3.Title	1.Study Design<br>2.Type of study<br>3.Country<br>4.Setting of the study	1.Sample Size (Maternal, Infant)<br>2.Maternal Age (Mean/Range)<br>3. Infant Age (Mean/Range)<br>4.Parity<br>5.Socio economic status	1.Name of the Micronutrient(s) supplemented /deficient/studied:<br>2.Method of Assessment/ Supplementation<br>3.Timing of assessment<br>4. Prevalence of micronutrient deficiency<br>5. Identified factors responsible for micronutrient deficiency	1.Outcome Domains (e.g., cognitive, motor, language)<br>2.Measurement Tools Used<br>3.Timing of Assessment (infant age at evaluation)	1.Statistical Analysis<br><br>2.Confounding variables adjusted				

Preferred Reporting Items for Systematic reviews and Meta-Analyses extension for Scoping Reviews (PRISMA-ScR) Checklist

SECTION	ITEM	PRISMA-ScR CHECKLIST ITEM	REPORTED ON PAGE #
**TITLE**			
Title	1	Identify the report as a scoping review.	1
**ABSTRACT**			
Structured summary	2	Provide a structured summary that includes (as applicable): background, objectives, eligibility criteria, sources of evidence, charting methods, results, and conclusions that relate to the review questions and objectives.	1,2
**INTRODUCTION**			
Rationale	3	Describe the rationale for the review in the context of what is already known. Explain why the review questions/objectives lend themselves to a scoping review approach.	4,5
Objectives	4	Provide an explicit statement of the questions and objectives being addressed with reference to their key elements (e.g., population or participants, concepts, and context) or other relevant key elements used to conceptualize the review questions and/or objectives.	5,6
**METHODS**			
Protocol and registration	5	Indicate whether a review protocol exists; state if and where it can be accessed (e.g., a Web address); and if available, provide registration information, including the registration number.	NA
Eligibility criteria	6	Specify characteristics of the sources of evidence used as eligibility criteria (e.g., years considered, language, and publication status), and provide a rationale.	10,11
Information sources*	7	Describe all information sources in the search (e.g., databases with dates of coverage and contact with authors to identify additional sources), as well as the date the most recent search was executed.	9,10
Search	8	Present the full electronic search strategy for at least 1 database, including any limits used, such that it could be repeated.	7, 8 and Appendix 1
Selection of sources of evidence†	9	State the process for selecting sources of evidence (i.e., screening and eligibility) included in the scoping review.	10,11,12
Data charting process‡	10	Describe the methods of charting data from the included sources of evidence (e.g., calibrated forms or forms that have been tested by the team before their use, and whether data charting was done independently or in duplicate) and any processes for obtaining and confirming data from investigators.	14 and Appendix 3
Data items	11	List and define all variables for which data were sought and any assumptions and simplifications made.	13
Critical appraisal of individual sources of evidence§	12	If done, provide a rationale for conducting a critical appraisal of included sources of evidence; describe the methods used and how this information was used in any data synthesis (if appropriate).	14
Synthesis of results	13	Describe the methods of handling and summarizing the data that were charted.	14,15

St. Michael's
Inspired Care.
Inspiring Science.

SECTION	ITEM	PRISMA-ScR CHECKLIST ITEM	REPORTED ON PAGE #
**RESULTS**			
Selection of sources of evidence	14	Give numbers of sources of evidence screened, assessed for eligibility, and included in the review, with reasons for exclusions at each stage, ideally using a flow diagram.	16 and final and complete numbers will be givne in
Characteristics of sources of evidence	15	For each source of evidence, present characteristics for which data were charted and provide the citations.	will be done in scoping review
Critical appraisal within sources of evidence	16	If done, present data on critical appraisal of included sources of evidence (see item 12).	will be done in scoping review
Results of individual sources of evidence	17	For each included source of evidence, present the relevant data that were charted that relate to the review questions and objectives.	will be done in scoping review
Synthesis of results	18	Summarize and/or present the charting results as they relate to the review questions and objectives.	will be done in s
**DISCUSSION**			
Summary of evidence	19	Summarize the main results (including an overview of concepts, themes, and types of evidence available), link to the review questions and objectives, and consider the relevance to key groups.	16,17,18 and will be done in scoping review
Limitations	20	Discuss the limitations of the scoping review process.	17
Conclusions	21	Provide a general interpretation of the results with respect to the review questions and objectives, as well as potential implications and/or next steps.	will be done in scoping review
**FUNDING**			
Funding	22	Describe sources of funding for the included sources of evidence, as well as sources of funding for the scoping review. Describe the role of the funders of the scoping review.	20

JBI = Joanna Briggs Institute; PRISMA-ScR = Preferred Reporting Items for Systematic reviews and Meta-Analyses extension for Scoping Reviews.
* Where *sources of evidence* (see second footnote) are compiled from, such as bibliographic databases, social media platforms, and Web sites.
† A more inclusive/heterogeneous term used to account for the different types of evidence or data sources (e.g., quantitative and/or qualitative research, expert opinion, and policy documents) that may be eligible in a scoping review as opposed to only studies. This is not to be confused with *information sources* (see first footnote).
‡ The frameworks by Arksey and O'Malley (6) and Levac and colleagues (7) and the JBI guidance (4, 5) refer to the process of data extraction in a scoping review as data charting.
§ The process of systematically examining research evidence to assess its validity, results, and relevance before using it to inform a decision. This term is used for items 12 and 19 instead of "risk of bias" (which is more applicable to systematic reviews of interventions) to include and acknowledge the various sources of evidence that may be used in a scoping review (e.g., quantitative and/or qualitative research, expert opinion, and policy document).

From: Tricco AC, Lillie E, Zarin W, O'Brien KK, Colquhoun H, Levac D, et al. PRISMA Extension for Scoping Reviews (PRISMAScR): Checklist and Explanation. Ann Intern Med. 2018;169:467–473. doi: 10.7326/M18-0850.
