# Supplementary material for: Maternal Micronutrient Status During Pregnancy and Its Neurodevelopmental Implications for Infants in South Asia: Protocol for a Scoping Review
Source: JMIR Res Protoc. 2025 Dec 15;14:e81592. doi: 10.2196/81592 (PMC12705126; doi:10.2196/81592)
Supplement: Multimedia Appendix 2 [file resprot-v14-e81592-s002.docx]

| **Draft table for electronic database search history** | | | | |
| --- | --- | --- | --- | --- |
| **Date of search** | **Electronic database** | **Keywords searched** | **Number of studies retrieved** | **Number of studies selected** |
|  |  |  |  |  |
|  |  |  |  |  |
|  |  |  |  |  |
|  |  |  |  |  |
