# Supplementary material for: Maternal Micronutrient Status During Pregnancy and Its Neurodevelopmental Implications for Infants in South Asia: Protocol for a Scoping Review
Source: JMIR Res Protoc. 2025 Dec 15;14:e81592. doi: 10.2196/81592 (PMC12705126; doi:10.2196/81592)
Supplement: Multimedia Appendix 3 [file resprot-v14-e81592-s003.docx]

Screening Forms

**Study Selection Form for Title/Abstract Screening**

1. **General Study Information**

| **Study Title** |  |
| --- | --- |
| **First Author** |  |
| **Year of Publication** |  |
| **Name of the Journal** |  |
| **Abstract Available** |  |

**B) Screening Criteria**

1. **Language:** Title/abstract in English  
   □ Yes    
   □ No
2. **Subjects:** Study includes human participants  
   □ Yes

□ Unclear   
□ No

1. **Study Population:** Involves healthy pregnant women  
   □ Yes

□ Unclear   
□ No

1. **Study Population:** Involves full-term infants (age < 2 years)

 □ Yes

 □ Unclear

 □ No

1. **Micronutrient Status:** Mentions maternal micronutrient status/intake/supplementation  
   □ Yes

□ Unclear   
□ No   

1. **Neurodevelopmental Outcomes:** – Refers to infant neurodevelopment (e.g., cognitive, motor, language outcomes)  
   □ Yes

**□** Unclear   
□ No

1. **Association Established:** – Association of Micronutrient Status with Infant Neurodevelopment

      □ Yes

**□** Unclear   
                  □ No

**STATUS OF INITIAL SCREENING**

**□** Include (All 7 criterion questions checked "Yes" or "Unclear")  
□ Exclude (Any of the 7 criteria questions checked "No")

**Reason for Exclusion: ________________________________________**

***Reasons for Exclusion**

1. Language other than English

2. Animal studies

3. Wrong population

3A. Either of one population i.e., pregnant women or infants is missing). Exclusion Women with multiple pregnancies and adolescent pregnancies or

3B. Pregnant females with diagnosed chronic illnesses, or infants born extremely pre-term, severe SGA and Neonatal Neural/congenital Disorders)

4. Wrong outcome (No Association of Micronutrient Status with Infant Neurodevelopment)

**Study Selection Form for Full-Text Screening**

1. **General Study Information**

| **Study Title** |  |
| --- | --- |
| **First Author** |  |
| **Year of Publication** |  |
| **Name of the Journal** |  |
| **Name of Country** |  |

**B) Screening Criteria**

1. Is Full Text available?  
          □ Yes    
          □ No

1. Is Full Text available in English?

□Yes  
□ No

1. Is the age of the pregnant women between 15-49 years?  
          □ Yes   
          □ No

1. Is the age of the infant ≤ 2 years?  
          □ Yes   
          □ No

1. **Micronutrient Status:** Does the study assess maternal micronutrient status/ micronutrient dietary intake/micronutrient supplementation during pregnancy? *(biochemical assessment)*

□Yes  
□ No

1. **Neurodevelopmental Assessment:** Does the study do neurodevelopmental assessment of infants (≤ 2 years)?

□Yes  
□ No

1. **Association Established:** Has the study established a clear relationship linking maternal micronutrient status with infant neurodevelopment?

□Yes  
□ No

**C) Study Population Exclusion Criteria**

1. Is the study conducted **exclusively on adolescent pregnancies** or **multiple gestations**?  
       □Yes   
       □ No

1. Does the study enrolled **pregnant women with pre-existing medical conditions** (e.g., chronic diseases, mental health disorders, substance abuse, or undergoing infertility treatment)?  
       □Yes   
       □ No

1. Is the study conducted **exclusively on infants born extremely preterm**, **severely small for gestational age (SGA)**, or with **pre-diagnosed neural/congenital disorders**?  
       □Yes   
      □ No

**STATUS OF FINAL SCREENING**

- **Include** (If   questions 2- 7 checked "Yes")
- **Exclude** (Any of the question from 2-7 checked "No" OR any of the questions from 8- 10 checked Yes)
- **Pending**
- (If Q1 answers No)
- (Closely related study so will be considered for snowballing)

**Reason for Exclusion: ________________________________________**

***Reasons for Exclusion**

1. Language other than English

2. 2A Pregnant Women aged < 15 years or > 49 years.

  2B Women with multiple pregnancies and adolescent pregnancies or pregnant females with diagnosed chronic illnesses, or infants born extremely pre-term, severe SGA and Neonatal Neural/congenital Disorders.

3. Wrong outcome (No Association of Micronutrient Status with Infant Neurodevelopment)

9. Not Applicable
