# Supplementary material for: Maternal Micronutrient Status During Pregnancy and Its Neurodevelopmental Implications for Infants in South Asia: Protocol for a Scoping Review
Source: JMIR Res Protoc. 2025 Dec 15;14:e81592. doi: 10.2196/81592 (PMC12705126; doi:10.2196/81592)
Supplement: Multimedia Appendix 4 [file resprot-v14-e81592-s004.docx]

Data Extraction Form

| **Data extraction Form Search 1 conducted on global basis** | | | | | | | | | | |
| --- | --- | --- | --- | --- | --- | --- | --- | --- | --- | --- |
| **S. No** | **Study**  **Identification** | **Study Characteristics** | **Population**  **Characteristics** | **Exposure/**  **Intervention Details** | **Infant**  **Neurodevelopmental**  **Outcomes** | **Statistical Considerations** | **Key Findings (Factors)** | **Grading and Strength of Evidence** | **Author Reported Limitations** | **Fundings** |
|  | 1.Author(s)  2.Year of Publication  3.Title | 1.Study Design  2.Type of evidence source  3.Country  4.Setting of the study | 1.Sample Size  (Maternal, Infant)  2.Maternal Age (Mean/Range)  3. Infant Age (Mean/Range)  4.Parity  5.Socio economic status | 1.Name of the Micronutrient(s) supplemented /deficient/studied:  2.Method of Assessment/ Supplementation  3.Timing of Exposure/Intervention  a. Dosage and frequency of supplementation  b. Duration of supplementation  4. Other intervention Specifics | 1.Outcome Domains (e.g., cognitive, motor, language)  2.Measurement Tools Used  3.Timing of Assessment (infant age at evaluation) | 1.Statistical Analysis    2.Confounding variables adjusted |  |  |  |  |

| **Data extraction Form for Search 2 focused on South Asian Countries** | | | | | | | | | | |
| --- | --- | --- | --- | --- | --- | --- | --- | --- | --- | --- |
| **S. No** | **Study**  **Identification** | **Study Characteristics** | **Population**  **Characteristics** | **Exposure/**  **Intervention Details** | **Infant**  **Neurodevelopmental**  **Outcomes** | **Statistical Considerations** | **Key Findings (Factors)** | **Grading and Strength of Evidence** | **Author Reported Limitations** | **Fundings** |
|  | 1.Author(s)  2.Year of Publication  3.Title | 1.Study Design  2.Type of study  3.Country  4.Setting of the study | 1.Sample Size  (Maternal, Infant)  2.Maternal Age (Mean/Range)  3. Infant Age (Mean/Range)  4.Parity  5.Socio economic status | 1.Name of the Micronutrient(s) supplemented /deficient/studied:  2.Method of Assessment/ Supplementation  3.Timing of assessment  4. Prevalence of micronutrient deficiency  5. Identified factors responsible for micronutrient deficiency | 1.Outcome Domains (e.g., cognitive, motor, language)  2.Measurement Tools Used  3.Timing of Assessment (infant age at evaluation) | 1.Statistical Analysis    2.Confounding variables adjusted |  |  |  |  |
