# Supplementary material for: Maternal Micronutrient Status During Pregnancy and Its Neurodevelopmental Implications for Infants in South Asia: Protocol for a Scoping Review
Source: JMIR Res Protoc. 2025 Dec 15;14:e81592. doi: 10.2196/81592 (PMC12705126; doi:10.2196/81592)
Supplement: Peer Review Report 1 [file resprot-v14-e81592-s006.pdf]

NIHR | National Institute for Health and Care Research

NIHR Coordinating Centre
Grange House
15 Church Street
Twickenham
TW1 3NL

Tel: 020 8843 8000
Email: ghrdevelopmentawards@nihr.ac.uk
www.nihr.ac.uk

Dr Jitender Nagpal
By Email

08 August 2024

Dear Dr Nagpal,

NIHR Global Health Research Development Awards: NIHR208259 - A UK-South Asia Collaborative to address the impact of maternal micronutrient deficiencies on subsequent infant-neurodevelopment

I am pleased to inform you that the Department of Health and Social Care, in their capacity as the National Institute for Health Research (NIHR), has confirmed their intention to award funding to your Development Awards Call 1 application. The intention to award funding is conditional upon acceptance of the terms and conditions set out in the NIHR ODA (Official Development Assistance) standard research contract and pending agreement to the suggested amendments recommended by the Development Awards Funding Committee and any Financial and Intellectual Property (IP) queries, as detailed in the Annex.

The NIHR Global Health Research Development Awards Short Form Funding Agreement between Contractors and the Secretary of State for Health and Social Care can be found on the NIHR Global Health Contracting webpage.

Next Steps

The NIHR is committed to the rapid initiation of research following the decision to fund. Therefore, we expect funded researchers to be working towards gaining the necessary contractual agreements and governance approval required to start the project on 01 January 2025.

Prior to contracting, you will be required to undergo a Due Diligence assessment.

The NIHR acknowledges the risk to organisations around committing resources to research before a contract is in place; however, it is rare to not reach contractual terms unless the circumstance of the research team changes. The NIHR, therefore, encourages organisations to commit staff to setting up projects at as early an opportunity as possible in order to expedite the formal commencement of research.

It is acknowledged that there can be unforeseen delays in starting up a research project, but in order to help reduce these it is your responsibility to work closely with your organisation’s R&D department or equivalent as well as other colleagues/departments/institutions involved in the administration and management of the research, and to start these discussions at the earliest opportunity.

To ensure that the project starts within the agreed timeframe with all the required agreements and approval in place, appropriate staff (such as project and/or study managers) need to be in post as early as possible after receiving this letter of intent. These staff costs will ultimately be covered through the research funding award.

To support the often-iterative process towards agreement of the contract, we have set out the guiding timeframes for the submission of responses or information for each step towards the agreement of the Development Awards Short Form Funding Agreement as well as the anticipated contract signature date.

- Confirmation of acceptance of funding – within 2 weeks from date of Intent to Fund Letter.
- Responses to Funding Committee feedback – within 3 weeks of receipt of the Intent to Fund Letter.
- Responses to Finance and IP queries – within 3 weeks of receipt of the Intent to Fund Letter.
- Submission of contracting institution due diligence documentation - within 3 months of receipt of the Intent to Fund Letter.
- Submission of Project-Specific Risk Register- within 1 month of receipt of Intent to Fund Letter
- Contract signature – by 31 December 2024
- Submission of Downstream Partner Due Diligence Requirements - within 3 months of Project Start Date
- Submission of Draft Collaboration Agreements and/or subcontracts (where applicable)- within 3 months of Project Start Date
- Submission of Signed Collaboration Agreements and/or subcontracts (where applicable)- within 6 months of Project Start Date

On receipt of information as set out above, the NIHR is committed to responding to your submission of information within four weeks or we will update you on progress.

No publicity concerning this award should be undertaken prior to completion of contracts for this award and for all other awards as part of Development Awards Call 1, and without full involvement of NIHR's Communications team. Please liaise with your NIHR Specific Point of Contact (SPOC) in the first instance on any issues relating to publicity.

Please take the time to carefully read the enclosures to this letter which details the feedback on your application, your contact within the NIHR who will be working with you on the contract, the processes to be undertaken during the next steps, as well as additional information relating to your award.

Yours sincerely,

Bianca De Bortoli
Senior Programme Manager

R&D Contact cc: Neetu Bansal (neetu.bansal14@gmail.com)

ANNEX

Contact details of your nominated NIHR SPOC

SPOC: Ianina Conte

E: ianina.conte@nihr.ac.uk

Feedback from Funding Committee

- The Funding Committee considered the application to be relevant to the call and in scope.
- The team was considered to be experienced with a demonstrable track record.
- The Community Engagement and Involvement (CEI) plans and activities were well received by the Funding Committee.
- The application was considered to offer good value for money.
- There was a clear plan to lead to a larger competitive funding proposal.
- The Funding Committee, however, raised the following:
    - While collaborators in India and Nepal have been clearly outlined, emerging partnerships within the remaining countries (Bangladesh, Bhutan and Sri Lanka) lacked detail.
    - While the capacity development plans for community members and early career researchers were well described in the application, institutional capacity development plans lacked detail.
    - The Plain English Summary was not considered suitable for a lay audience.
    - The application lacked detail on the rationale for using a mixed methods approach to understand micronutrient deficiencies (Workstream 2) (in terms of research design, sampling, tools and analytical methods) and the rationale for the chosen sample size in the quantitative analysis plan. It was unclear how the team members plan to triangulate their findings.

The Funding Committee agreed that the application met the necessary competitiveness standard and should be recommended for funding.

Note on ODA eligibility: No concerns had been raised regarding ODA eligibility during pre-panel committee checks or the panel committee discussion.

Finance queries

- The Indian government has recently updated the Foreign Contribution Regulation Act (FCRA) which relates to payments to organisations in India. These organisations should follow the latest Indian government guidelines to confirm whether they require a FCRA license and that they have the appropriate measures in place to receive funding. It is

the Contracting Organisation’s responsibility to ensure that any organisations in India involved in the research proposal, whether as Lead Applicants, Co-Applicants or Collaborators, are fully compliant with the requirements of the FCRA. They should also ensure that the organisation continues to be compliant with FCRA requirements for the duration of the award.

- Specific finance questions:
    - Please provide further breakdown of the cost “International & local” related to Travel, Subsistence & Conference costs for £16,346.
    - Please provide further breakdown of the consumables cost for conducting workshops (£4,279).
    - Please remove the costs for the research advisor from QMUL (£9,615) as salary costs can only be claimed for individuals based in / employed by LMIC organisations.

IP queries

- Intellectual property (IP) positions are prescribed in Section 7 of the GHR Development Awards Short Form Funding Agreement (.DOCX - Word). For the GHR Development Awards, IP will be owned by the contracting organisation and Joint Ownership of IP is not permitted. Acknowledgement of this position is integral funding acceptance.
